# Investigating the structure of the greylag goose vocal repertoire: what can unsupervised methods tell us?

**DOI:** 10.1038/s41598-026-51404-2

**Published:** 2026-06-03

**Authors:** Lena Gies, Jonas Lesigang, Sonia Kleindorfer, Tecumseh Fitch

**Affiliations:** 1 Department of Behavioral and Cognitive Biology, University of Vienna, Vienna, Austria; 2 Konrad Lorenz Research Center for Behaviour and Cognition, University of Vienna, Grünau im Almtal, Austria

**Keywords:** Greylag goose, Vocal repertoire, Clustering, Representation, Bioacoustics, Autoencoder, Computational biology and bioinformatics, Mathematics and computing, Zoology

## Abstract

Defining a comprehensive signal repertoire is an important step to understanding a species’ vocal communication system. Here, we investigated the vocal repertoire of a well-investigated model species in ethology: the greylag goose (*Anser anser*). We applied unsupervised machine learning algorithms to a large dataset of vocalisations from a free-living population of greylag geese to investigate the acoustic structure of this species’ vocal signals. We extracted four types of data representations, which were projected into 2, 20 and 100 dimensions using the UMAP algorithm, and then grouped using two commonly used clustering methods. Additionally, we successfully applied a graph-based clustering approach — Leiden community detection — which, to our knowledge, has not previously been employed in bioacoustics. Our analyses revealed a partly graded vocal repertoire that broadly matched early descriptions of the greylag goose call repertoire. Audio feature vectors, rather than more commonly used spectrographic representations, revealed clusters most congruent with human labels and offered the most comprehensive visualisation of the acoustic space. Leiden community detection performed comparably to established approaches but matched the number of human-defined classes closest. These findings highlight the impact of data representation on repertoire analysis and provide the first objective, quantitative characterisation of the greylag goose vocal repertoire.

**Supplementary Information:**

The online version contains supplementary material available at 10.1038/s41598-026-51404-2.

## Introduction

The study of non-human communication systems presents a multitude of challenges, starting with the definition of their signal categories. While trying to understand these systems, for which first-hand knowledge and insight into the species-typical *Umwelt*^1^(the sensory-motor world of the individual) is limited, researchers have traditionally drawn conclusions based on extensive behavioural observations, which are highly dependent on our own sensory biases and perspectives. This subjective element increases the likelihood of observer bias^2^, and partial or invalid conclusions. Manually scanning large amounts of data can reduce bias to some extent^3^ but can require impractical amounts of time. As acoustic datasets continue to grow in size and complexity, there is an increasing need for analytical tools that can systematically explore large amounts of data while reducing observer bias. Modern machine learning (ML) approaches provide a promising solution, potentially allowing less biased and more comprehensive explorations of large datasets of animal vocalizations.

In recent years, ML methods have been increasingly used to study non-human animal communication to determine signal classes, with a strong emphasis on the vocal modality. The term ML covers a range of analytical approaches that have proven useful for analysing bioacoustic data^4–6^. For example, Martin and colleagues^4^ combined latent projections derived from a distance matrix of calls with hierarchical clustering, to analyse a large dataset of rook vocalisations (*Corvus frugilegus*), revealing sex-specific vocal repertoires. Elie and Theunissen^5^ analysed the zebra finch (*Taeniopygia guttata*) vocal repertoire using four different data representations combined with supervised and unsupervised algorithms, allowing detailed quantification of call-type structure. Goffinet and colleagues^6^ showed that features extracted from the latent space of a variational autoencoder (VAE, a deep learning approach to encoding and decoding data^7^ trained on spectrograms outperform manually selected acoustic features when analysing zebra finch and mouse (*Mus musculus*) vocalisations. Similarly, Best and colleagues reported improved congruence with human labels across datasets from six species using a convolutional autoencoder^8^. However, as with any analytical tool, ML methods only provide meaningful results when applied to appropriate questions with suitable data representations.

The outcome of ML analyses may depend on the representation of the data, the size of the dataset, and the selected parameters and algorithm^8–11^. Sainburg, Thielk, and Gentner^9^ reviewed common dimensionality reduction and clustering algorithms, identifying combinations of Principal Component Analysis (PCA) and Uniform Manifold Approximation and Projection (UMAP), which reduce the number of dimensions of the data, with Hierarchical Density-Based Spatial Clustering of Applications with Noise (HDBSCAN), a hierarchical clustering algorithm, to produce clusters best matching hand-labelled call classes in two songbird species. However, only few studies have examined how data preprocessing and representation (e.g., spectrograms vs. acoustic signals) influence model performance in bioacoustics^8,11–15^. These studies show that representation learning and unsupervised feature extraction can substantially improve classification and clustering performance compared with manually selected acoustic features^8,11,14,16^. These studies highlight that the effects of different representations remain insufficiently explored across datasets and analytical approaches^13,14^. Here, we address this gap by systematically comparing multiple data representations and unsupervised clustering algorithms on a large dataset of greylag goose vocalisations.

Vocal communication in greylag geese (*Anser anser*) is a promising application area for ML methods. The species is highly social, vocally active, and has been a model system in ethology since the field’s early days^17^. Greylag geese are waterfowl of the family Anatidae, which have a simple vocal apparatus with a mostly straight trachea and only slight sexual dimorphism^18–20^: Greylag males typically vocalise at a higher pitch than females^19^. Anatomical studies and experiments with excised vocal apparatuses in other *Anser* species suggest that the tympaniform membranes provide the source of vocalisations in this genus^19^. Stress across the tympaniform membranes modifies fundamental frequency, and is related to the pressure in the clavicular air sac^19^. Multisyllabic calls have been found to have lower fundamental frequencies, ascribed to the pressure of the clavicular air sac being distributed over a longer time^19^. In addition to the fundamental frequency, formant frequencies (vocal tract resonances) can be adjusted via the tracheal musculature, which alters the length of the trachea^19,21^. These physiological mechanisms provide a basis for the acoustic variation observed within and between call types in the greylag goose vocal repertoire.

The vocal characteristics of greylag geese have been described impressionistically since the beginning of the 20th century. Heinroth^22^ first described seven different call types of the adult greylag goose in 1910: (1) The distance call, a loud, far-carrying call used when partners or families seek each other out without visual contact; (2) the contact call, a softer call produced in close proximity to the partner when foraging or walking; (3) the recruitment call (English name given by later authors^23^which resembles the contact call but shows a clearer syllabic structure and increased amplitude and is emitted before take-off, often accompanied by a distinct head-shaking behaviour (e.g^24^.); (4) the locomotion call, produced before traveling larger distances on foot^25^ (5) the alarm call, a harsh call often preceding flock movement toward nearby water; (6) hissing, a broadband non-vocal sound elicited when a goose is closely approached; and (7) the triumph call, a stereotyped call sequence produced when an individual returns to its mate after displacing a conspecific in the so-called triumph ceremony^24^. Ongoing long-term research has expanded these early descriptions, placing several adult call types on graded continua^24^ and suggesting a repertoire of roughly 11 call types^17,19,23,24,26^ with gradual transitions between some call types rather than strictly discrete categories.

Both experimental playback and more quantitative behavioural investigations into the species’ vocal identity have been conducted: Guggenberger and colleagues^27^ found that distance calls are individually distinctive, and are recognised by the caller’s partner in playback experiments. Weinhäupl^28^ found differing call characteristics between individuals’ departure calls as well as vocal differences between sexes. Lesigang^29^ extended these findings, identifying vocal identity signatures in the departure call using discriminant function analysis, and demonstrating vocal individual recognition in playback experiments. Körmer^30^ identified individual structural variation in affiliative contact calls. Lesigang and colleagues^31^ found that more contact calls were emitted during locomotion and by individuals with partners, with the partners responding to these calls more often than other members of the flock.

Here, we aimed to quantitatively investigate the structure of the greylag goose vocal repertoire using modern ML approaches. We extracted four types of data representations from a manually labelled dataset of single-syllable vocalisation segments: (1) a set of 23 audio feature vectors, (2) linear frequency-scaled spectrograms, (3) linear frequency cepstral coefficients (LFCC), which quantify the overall shape of the spectrogram, and (4) the latent vector of a convolutional variational autoencoder (VAE). The dimensionality of all four representation types was reduced to 2, 20 or 100 dimensions using UMAP, before clustering the data with two common approaches: k-means, a non-hierarchical centroid-based clustering algorithm, and the hierarchical density-based HDBSCAN algorithm. Additionally, we applied Leiden community detection, a graph-based algorithm not previously used for vocal repertoire analyses, to the nearest-neighbours graph directly, allowing data clustering without dimension reduction. We compared the resulting clusters with human-assigned call classes in terms of the number of detected classes and the assignment of calls to clusters. We also examined whether the acoustic space shows a graded structure, consistent with earlier descriptions of the greylag goose vocal repertoire. Finally, we explore how different data representations and clustering approaches influence the inferred structure of the repertoire.

## Results

### Repertoire structure

**Fig. 1:**
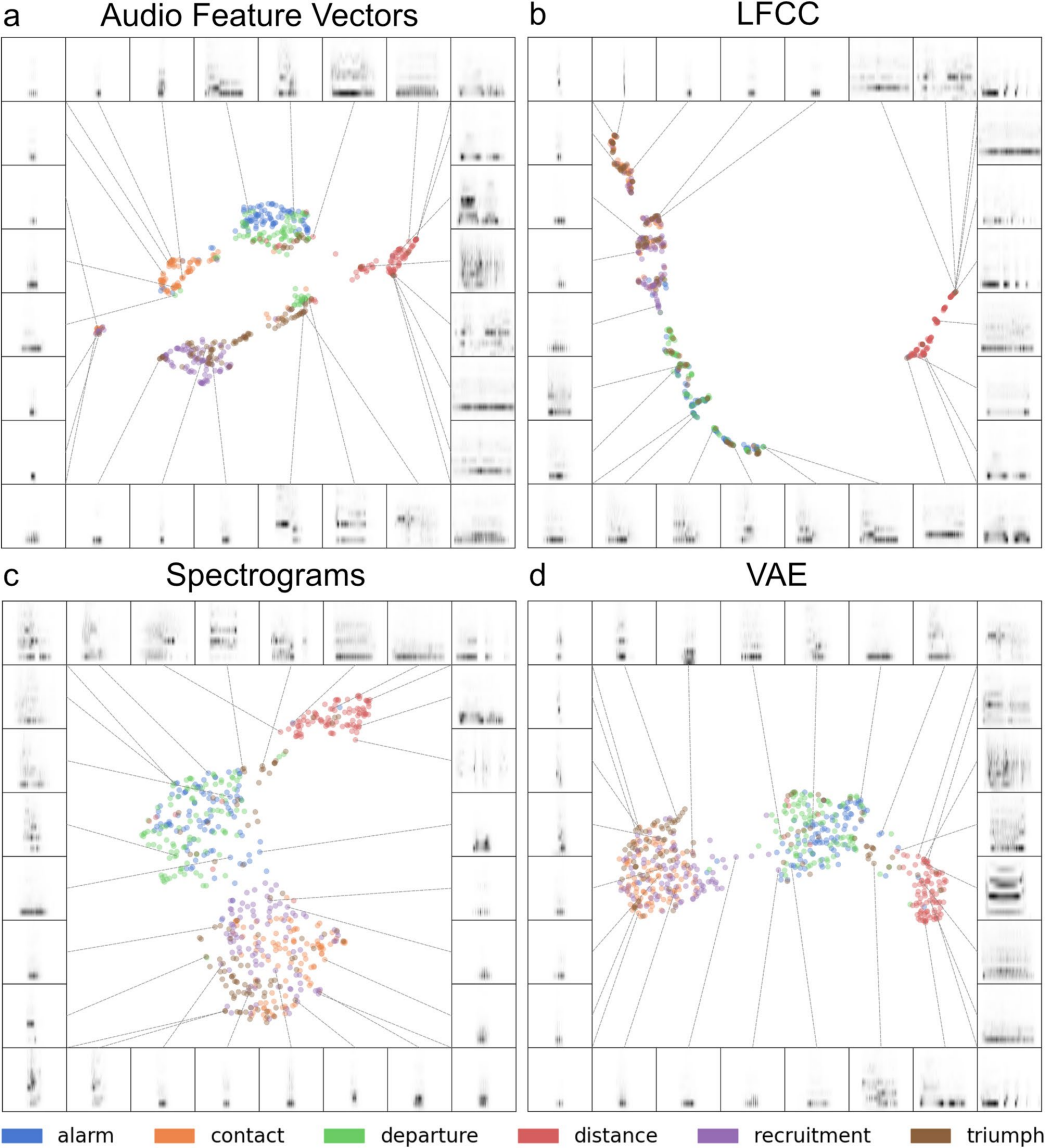
Example UMAP projections of the audio segments based on different representations with subset sizes of up to 100 calls per class. Colours represent human-labelled class identities. (**a**) Embeddings of audio feature vectors. (**b**) Embeddings of linear frequency cepstral coefficients. (**c**) Embeddings of log-padded spectrograms. (**d**) Embeddings of variational autoencoder latent vectors.

All four data representation types exhibited Hopkins scores indicating non-random distributions (range 0 to 1, with higher likelihood random distribution at 0). Hopkins scores for each representation were as follows: audio feature vectors: 0.97 ± 0.02, LFCC: 0.99 ± 0.01, spectrograms: 0.93 ± 0.05, VAE latent vectors: 0.94 ± 0.03 ($\:H\pm\:$ SD). While the visualizations appear very different, several regularities are present: Sample embeddings from the contact and recruitment call classes substantially overlapped in all representations except for audio feature vectors (Fig. 1). Similarly, audio segments labelled as triumph calls spread over the groupings of contact and recruitment calls in the embedded space, with the exception of the call type ‘contact’ in embeddings of audio feature vectors, with which no overlap was generally visible. Distance calls were clearly distinctive in all embeddings, as was a subgroup consisting of the human-labelled departure and alarm call classes. LFCC- and audio feature vector-based embeddings revealed individual vocal signatures, most prominently in distance calls but visible in other call type classes as well (see Figures S1–S5). The sexual vocal dimorphism previously described^18–20^ in the literature was visible in call type classes with sufficient data: Distance, contact, and recruitment call groupings showed a gradual split of the sexes (see Figure S6).

### Call type classification

**Fig. 2:**
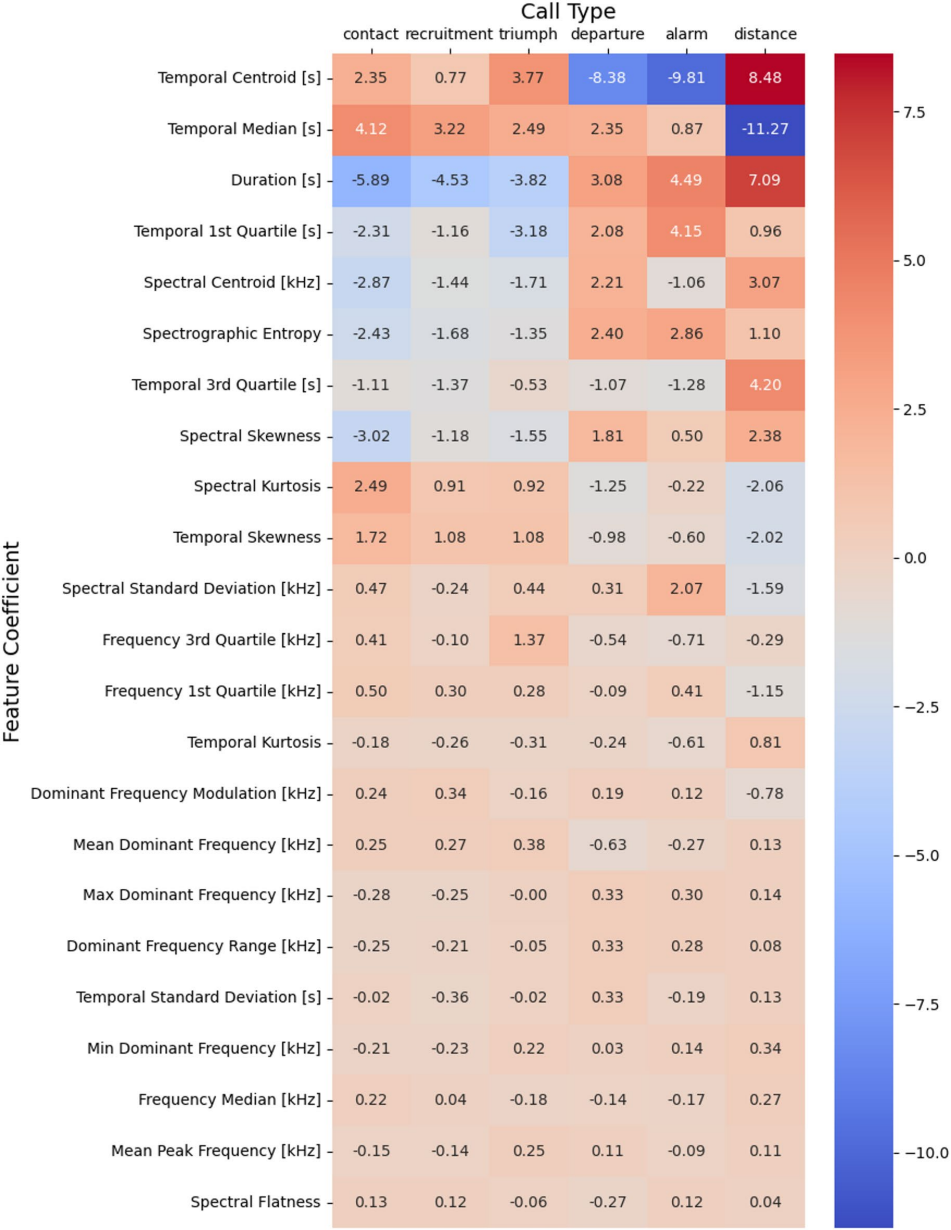
Feature coefficients per call type category obtained from LDA and sorted by variance.

To identify which acoustic features contribute most strongly to differences between call categories and to characterise the acoustic structure of the labelled repertoire, the corresponding audio feature vectors were classified using Linear Discriminant Analysis (LDA). This revealed that, of our 23 audio features, the call type classes exhibited the most variation in temporal features (temporal centroid, duration, temporal median, and first temporal quartile), as well as spectral centroid and spectrographic entropy. Figure 2 shows the feature coefficients of the fitted LDA sorted by variance (additionally, see Figures S7 and S8). The model had an overall acceptable performance with an f1 score of 0.60, which is significantly above chance (* p* = 0.002, permutation test, permuted 500 times). Class level f1 scores were as follows: alarm: 0.47 (support = 46), contact: 0.64 (support = 100), departure: 0.73 (support = 100), distance: 0.95 (support = 97), recruitment: 0.65 (support = 100), triumph: 0.48 (support = 51). LDA was unable to classify two classes: Only 37% of alarm calls were assigned correctly, whereas 50% were classified as departure calls. 41% of triumph calls were correctly labelled. The remaining triumph calls were assigned to the category ‘recruitment’ by 14%, ‘contact’ by 25%, and ‘departure’ by 20%. Moreover, ‘contact’ and ‘recruitment’ classes were interchanged at rates of 67% against 28% (true label: contact) and 68% against 30% (true label: recruitment). Variation in classification success across call types also reflects differences in within-class acoustic variability, which may influence how readily unsupervised clustering approaches recover these categories. For details see Supplementary Figure S9.

### Number of predicted call type classes

**Fig. 3:**
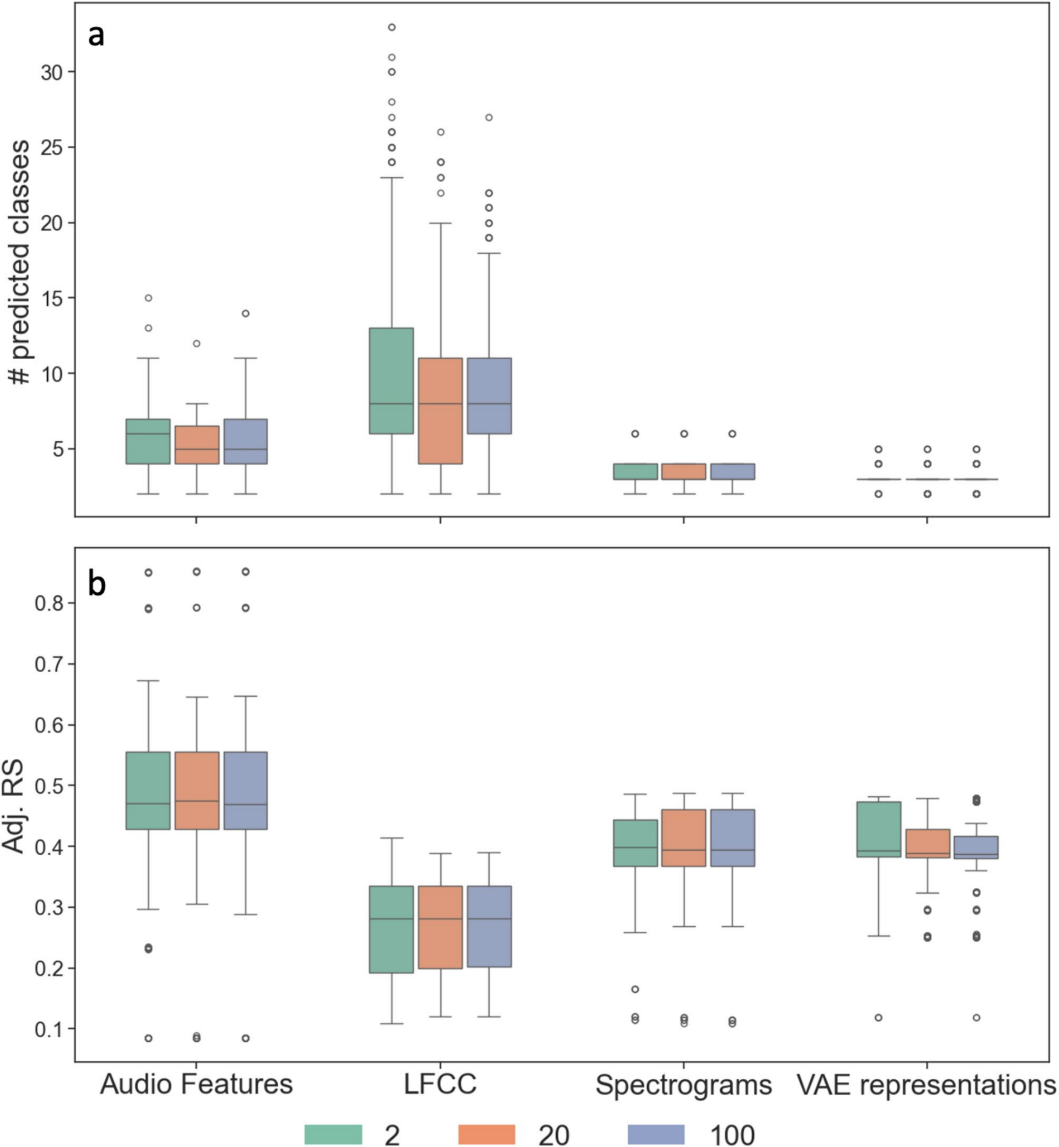
Predicted number of classes (**a**) and overlap with human labels (**b**, adjusted Rand score) for different data representation types, color coded for dimensionality. Although the number of dimensions did not have an effect on the results, we observed a tendency for reduced variability in the number of predicted classes with 20 and 100 dimensions and representations other than spectrograms and VAE vectors.

**Fig. 4:**
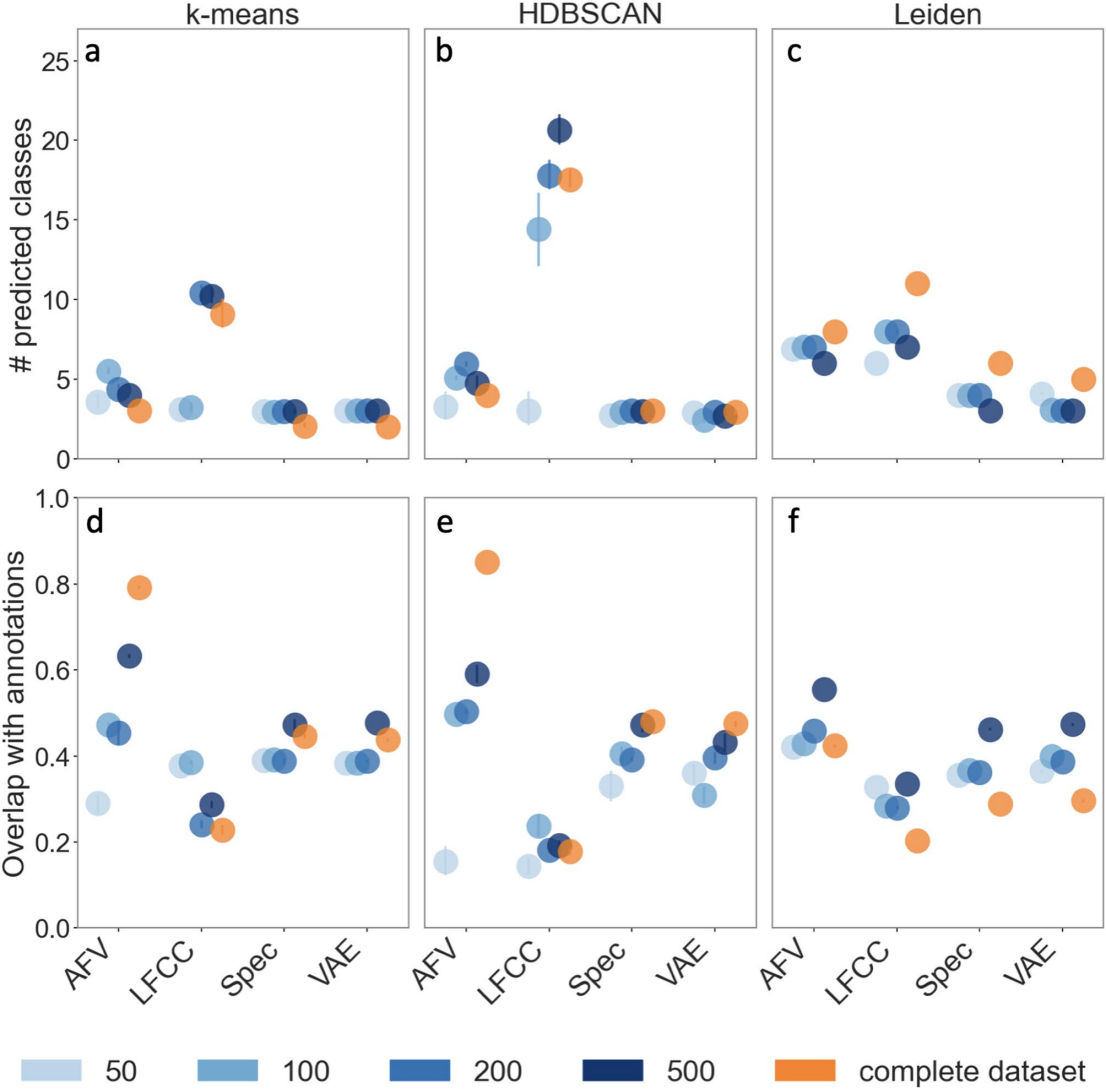
Number of predicted classes (**a**-**c**) and overlap of predicted classes with human annotations (**d**-**f**): Number of clusters and adjusted Rand scores with 95% confidence intervals for different data representation types and clustering algorithms. Colours represent maximum subset size. Alarm and triumph calls were excluded at a maximum subset size of 500. The entire dataset included class sizes ranging from 228 to 3214 and was bootstrapped five times, in contrast to 20 times with the randomly resampled subsets of predefined sizes. This was repeated for three dimensionalities: 2, 20 and 100.

When clustering the data embeddings using k-means, HDBSCAN and Leiden community detection algorithms, the overall mean number of detected classes was 5.26 ± 4.21 (mean ± SD, median: 3). The number of detected clusters was highly variable across algorithms for audio feature vectors and LFCC representations, ranging from 2 to 15 (audio feature vectors) and 33 (LFCC) classes, whereas the groupings from VAE vectors (2–5 clusters) and spectrograms (2–6 clusters) were less variable (Figs. 3 and 4, Table S1 and S2). Groupings detected by Leiden exhibited less variation (* n* = 5.38 ± 1.95, mean ± SD) across representations than those detected using HDBSCAN and k-means (HDBSCAN: * n* = 6.14 ± 6.40, mean ± SD, k-means: * n* = 4.25 ± 2.58). Clusterings based on audio feature vectors most closely matched the number of human labels overall, and produced high congruence between algorithms (k-means: * n* = 4.27 ± 1.26, HDBSCAN: * n* = 4.71 ± 1.98, Leiden: * n* = 6.80 ± 0.53, mean ± SD). When clustering LFCCs, k-means and HDBSCAN produced more variable results than predictions obtained from Leiden (k-means: * n* = 6.88 ± 3.80, HDBSCAN: * n* = 14.19 ± 8.42, Leiden: * n* = 7.46 ± 1.20, mean ± SD). Data represented through spectrograms and VAE vectors produced less variable results regarding the number of predicted classes across algorithms, but predicted fewer classes when compared to other representation types and the number of human labels (spectrograms: k-means: * n* = 2.91 ± 0.28, HDBSCAN: * n* = 2.92 ± 0.28, Leiden: * n* = 3.88 ± 0.68; VAE vectors: k-means: * n* = 2.94 ± 0.24, HDBSCAN: * n* = 2.75 ± 0.45, Leiden: * n* = 3.38 ± 0.63, mean ± SD). Independent of the data representation type, there was a tendency for higher variation in the number of predicted classes when clustering two-dimensional data representations (Fig. 3).

### Congruence between predicted and human-labelled classes

Silhouette scores of the clustered data, which measure how well clustered together datapoints of the same class are, exceeded zero for all representations: audio feature vectors: 0.66 ± 0.04, LFCC: 0.67 ± 0.04, spectrograms: 0.65 ± 0.07, VAE latent vectors: 0.72 ± 0.04 (*s* ± SD, range: − 1 to 1). Similarly, modularity values for communities detected using Leiden were as follows (range 0 to 1): audio feature vectors: 0.66 ± 0.02, LFCC: 0.74 ± 0.02, spectrograms: 0.51 ± 0.01, VAE latent vectors: 0.55 ± 0.02 ($\:Q\pm\:$ SD, also see Figure S10).

**Fig. 5:**
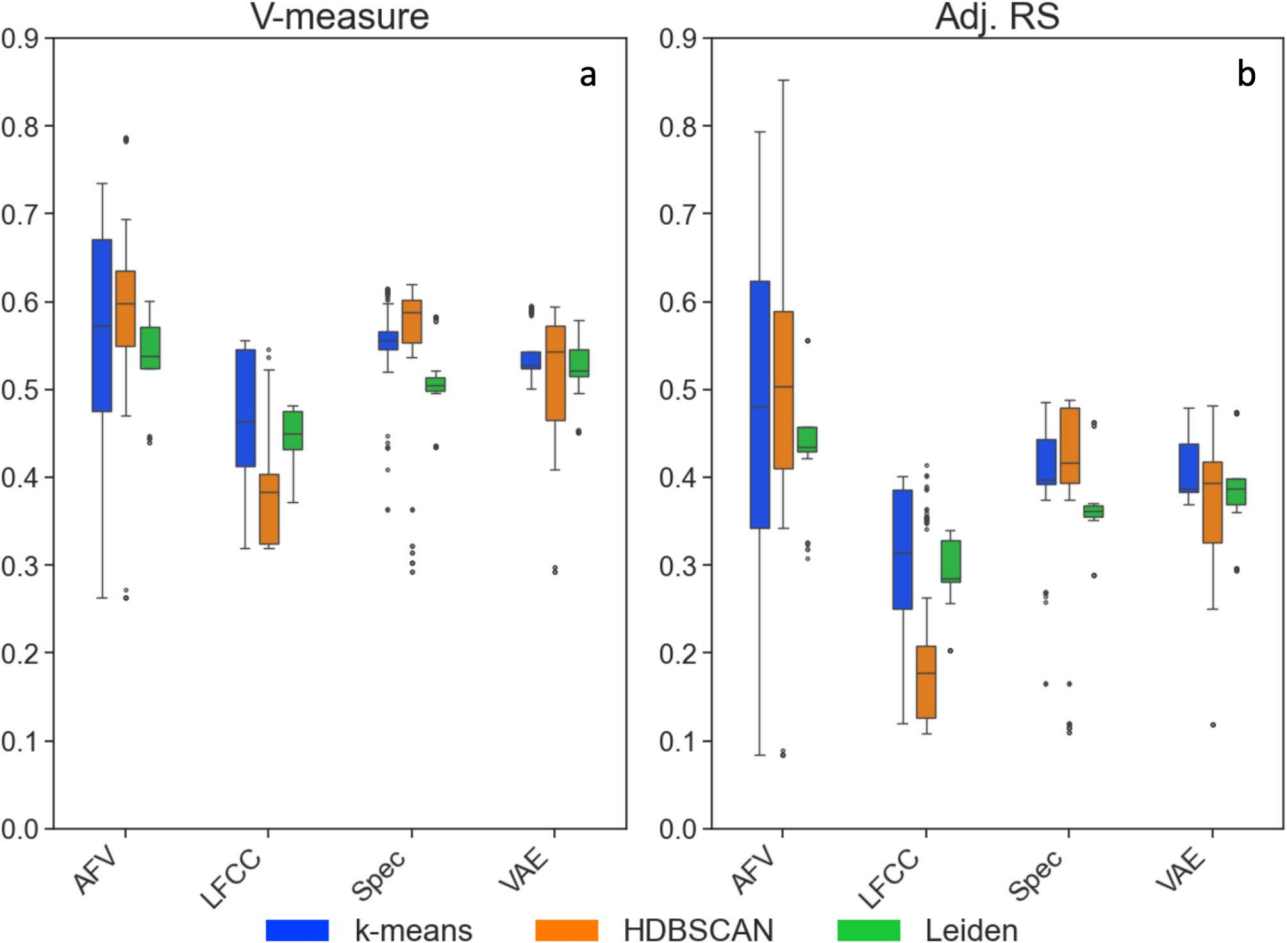
Overlap between human-labelled and detected class identities for different data representations and algorithms. Whiskers indicate data lying within 1.5 interquartile ranges of the upper and lower quartiles. The data was resampled 255 times in total with five different subset sizes for every representation and algorithm. V-measure scores (**a**) can range from zero to one, with one indicating a perfect overlap between true and predicted classes. Adjusted Rand score (**b**) ranges from − 0.5 to 1 with perfect alignment at a score of 1.

There was a significant effect on the overlap with human labels of data representation type (*p* < 0.001, χ^2^ = 1545.8), dataset size (*p* < 0.001, χ^2^ = 460.2), balance of the dataset (*p* < 0.001, χ^2^ = 318.2), and algorithm (V-measure, *p* < 0.001, χ^2^ = 112.7). Overlap between predicted and human-labelled classes was highest when using audio feature vectors (range 0 to 1, V-measure: 0.58 ± 0.12, range − 0.5 to 1, adj. RS: 0.50 ± 0.18) and spectrograms (V-measure: 0.54 ± 0.06, adj. RS: 0.40 ± 0.07; full results reported in Fig. 5 and Table S2 and S3). These metrics varied substantially depending on the subset sizes when using k-means and HDBSCAN to cluster audio feature vectors, whereas the overlap was less variable when detecting groups based on the nearest-neighbours graph directly using Leiden. However, congruence with human labels dropped when clustering the entire, unbalanced dataset using Leiden (Fig. 4). On the other hand, dataset size influenced the results less for spectrograms and VAE latent vectors when using HDBSCAN. Furthermore, the grouping overlap increased with bigger subset sizes when using k-means or HDBSCAN to cluster audio feature vectors. This led to the overall highest overlap with human labels when using HDBSCAN to cluster audio feature vectors using the entire dataset (V-measure ± SD: 0.79 ± < 0.01, adj. RS ± SD: 0.85 ± < 0.01). The dimensionality of the data did not influence the overlap of predicted and human-labelled classes (*p* = 0.637, χ^2^ = 0.22, Fig. 3 and Table S3).

**Fig. 6:**
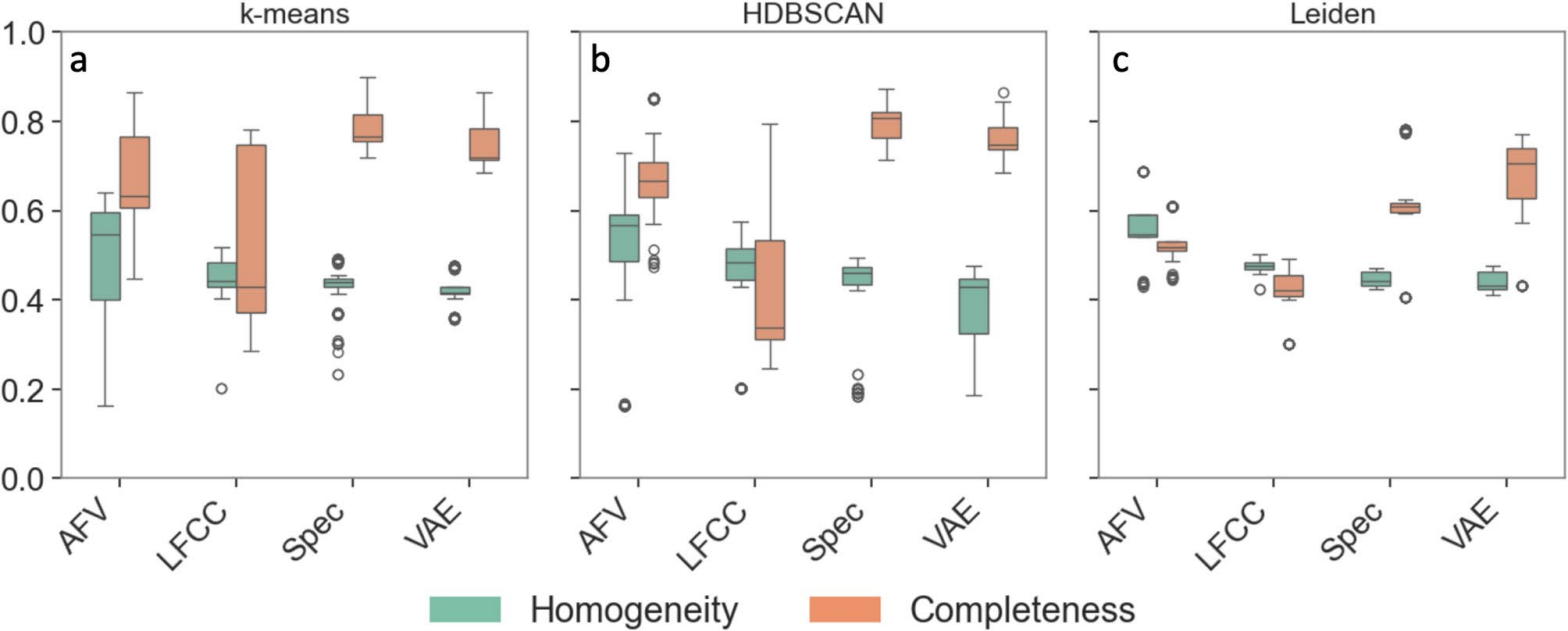
Homogeneity and completeness scores of predicted classes when compared to human annotations. Whiskers indicate data lying within 1.5 interquartile ranges of the upper and lower quartiles. The data was resampled 255 times in total with five different subset sizes for every representation and algorithm. (**a**) Classes predicted using the k-means algorithm. (**b**) Classes predicted using HDBSCAN. (**c**) Classes predicted using Leiden community detection.

The predicted classes had overall higher completeness than homogeneity values except for clusterings obtained through Leiden based on audio feature vectors and LFCCs (Fig. 6). High completeness is obtained when samples of the same human label mostly fall into the same predicted class, while high homogeneity is obtained when each predicted class mostly contains samples of a single human label.

## Discussion

We used unsupervised ML methods to investigate the vocal repertoire of the greylag goose. The visualisation of the acoustic space revealed a partly graded and overlapping vocal repertoire. Distance calls were clearly structurally distinct from other call types. A second distinct group of structurally similar alarm- and departure calls was found. Additionally, contact, recruitment and triumph calls occupied overlapping, but not identical, acoustic spaces. Crucially, automatically predicted class numbers as well as the overlap between clustered and human-labelled classes varied substantially for the different algorithms and data representations, indicating that methodological choices can have a major impact on ML-based evaluations of repertoire size and composition. Below, we first outline our conclusions regarding the different analysis methods and representations, and then turn to the structure of the greylag goose vocal repertoire.

### Methodological insights

To evaluate the performance of different machine learning methodologies, we compared their output to human call labels, which were made taking behavioral context into account. We found considerable differences in the structure of the embedded space, the number of predicted classes, and the congruence with human labels, depending upon the chosen data representation type, dataset size, and the algorithm chosen. Dimensionality did not have an effect on the congruence with human labels but using higher dimensionalities reduced variability in the number of predicted classes.

#### Audio feature vectors

Clusters based on audio feature vectors were most congruent with human labels, independent of the clustering algorithm used. In fact, the distinction between recruitment and alarm calls was *only* evident in embeddings based on audio features. This could be due to audio feature vectors mitigating noise in the data: The calls in this dataset have low tonality and limited frequency modulation when compared with the songs of songbirds or whistles of cetaceans, where spectrogram representations are the norm. Spectrogram-based representations might weigh frequency modulation, the tonality of the data, and the call length more than the spectral energy distribution. This skew might be balanced when using audio feature vectors as representations. This observation aligns with findings that audio feature vectors of zebra finch (*Taeniopygia guttata*) vocalisations encode more information than spectrograms^5^. The authors described zebra finch vocalisations as “broadband [with a] relatively restricted range of fundamental frequencies”, which also applies to greylag goose vocalisations. A study investigating Cassin’s vireo (*Vireo cassinii*) song found UMAP embeddings of spectrograms to be more distinctly clustered than those on audio feature vectors^9^, contrasting with our findings. The song of Cassin’s vireo is more tonal and less broadband than calls of the zebra finches or greylag geese. Overall, this suggests that audio feature vectors may be better suited to analyse broadband vocalisations than spectrograms, a hypothesis that should be investigated further with a larger number of species. This should include an investigation of the audio features used, as feature selection is an inherently biased process in itself.

#### Spectrogram-based representations

Among the spectrogram-based data representations, VAE latent vectors had the largest overlap with human labels, independent of the clustering algorithm. A consistent disparity was found between homogeneity and completeness for clusters based on VAE latent vectors, especially when compared with audio feature vector clusterings, indicating that VAE representations promote underclustering. The clusterings based on VAE latent vectors grouped human-labelled classes together into larger classes. When clustering spectrograms, classes predicted from HDBSCAN showed a small tendency to be more congruent with human labels than clusters obtained using all other algorithms, which is in line with findings by Sainburg and colleagues^9^: they found spectrograms embedded using UMAP and clustered with HDBSCAN to align better with human-labelled data than clusters obtained via k-means in two songbird species. Nevertheless, VAE latent vectors may be more appropriate when employing spectrogram-based representations to analyse vocalizations, as previously suggested by Goffinet and colleagues^6^, since they produced a higher congruence with human labels with less variable results. Again, more research on more species will be needed to resolve this apparent discrepancy.

#### Dataset size

Different balanced input subset sizes, or the use of the original unbalanced dataset, considerably influenced the number of automatically predicted clusters as well as the overlap between machine-clustered and human-labelled groups (Fig. 4). HDBSCAN predicted highly variable numbers of clusters and consequently varying V-measures for audio feature vectors, LFCCs, and spectrograms, but not VAE latent vectors, which were influenced the least overall. Groupings from k-means on representations other than LFCCs, as well as those based on Leiden with any of the representations, were more robust to different dataset sizes in terms of numbers of clusters. However, alignment with human labels dropped with the unbalanced original dataset in Leiden groupings based on representations other than audio feature vectors. Congruence with human labels increased with bigger dataset sizes when clustering audio feature vector embeddings in both k-means and HDBSCAN, reaching the highest overall V-measure with HDBSCAN applied to the original dataset. To conclude, although results from the combination of Leiden with audio feature vectors varied the least with different dataset sizes, with the largest dataset size better overlap with human labels was achieved using k-means or HDBSCAN, despite its unbalanced class distribution.

#### Clustering algorithms

Although Leiden produced comparable V-measures to the two other algorithms and the number of predicted classes was closest to those originally defined, the overlap with human labels decreased when using Leiden with spectrograms, in comparison to HDBSCAN. The use of audio feature vector embeddings to study vocal repertoires appears to be a sensible choice. Our results indicate that, for greylag geese, clustering audio feature vectors with HDBSCAN or k-means when the dataset is large, or with Leiden if less data is available, produces reasonable results. In some of the projections, Leiden was able to distinguish triumph and contact calls that were overlapping in the acoustic space, indicating it may be well suited for analysing graded signal repertoires (Fig. S11).

The large variation in the number of detected clusters across algorithms and data representations likely reflects methodological differences in (1) how cluster structure is defined and (2) the structure of the embeddings, rather than fundamentally different repertoire structures. In many cases, partitions with high cluster numbers appeared to represent subdivisions of broader acoustic groups identified by other methods (see Fig. S12). This pattern is expected because clustering algorithms differ in their sensitivity to local structure: density-based approaches such as HDBSCAN can split heterogeneous regions of acoustic space into multiple clusters if local density differences are present, whereas centroid-based approaches such as k-means may tend to merge such regions into broader categories, if clusters are non-convex.

In addition, variation in cluster numbers often arose from differences in the low-dimensional embeddings themselves. Different dataset sizes led to LFCC embeddings with varying numbers of visually distinguishable groupings (Figs. S11–S14), which then produced large numbers of predicted clusters. Graph-based approaches such as Leiden are less sensitive to such differences, as they do not rely on these embeddings, and produced more consistent estimates of cluster number.

Together, these results indicate that estimates of repertoire size derived from unsupervised clustering depend strongly on both the data representation and the analytical method, along with the size of the dataset. This finding necessitates a closer examination of how these differences correlate with the various parameters, which should additionally include other representation types and distance metrics.

### The greylag goose vocal repertoire

#### Repertoire structure

UMAP embeddings of the data revealed a partly graded vocal repertoire. This is supported by lower silhouette and modularity scores as well as low f1 scores for the triumph call category and confusion between recruitment and contact calls when classifying these call types using LDA. Contact and recruitment calls partly overlap in the acoustic space. This is most prominent in visual data representations and could be due to most weight being put on the call duration when embedding the visual data. This duration-dependence is moderated when embedding audio feature vectors, but is also apparent when investigating LDA coefficients further: When comparing the feature coefficients of the duration of all six call types, contact (− 5.89), recruitment (− 4.53), and triumph (− 3.82) do not differ as much from each other as they do to departure (3.08), alarm (4.49), and distance (7.09, see Fig. 2). The recruitment and contact call types differ mostly in entropy measurements, spectral skew, spectral kurtosis, temporal centroid, and duration, but otherwise share very similar feature coefficients. From personal observations, recruitment calls are generally louder than contact calls and have a more isochronous rhythm. This is not taken into account in our analysis which only analysed syllable-level segments, and should be quantitatively investigated in future research.

Similarly, departure and alarm calls share very similar acoustic structure, mostly differing in spectral centroid and temporal median, and visually overlap in all of the embeddings. This is reflected in a low f1 score when classifying alarm calls (but sample size was comparatively low). Triumph calls overlap with contact and recruitment calls but span into the departure and alarm call subgroup in terms of their acoustic structure. This supports Fischer’s^24^ hypothesis that calls emitted in the triumph ceremony fall into two graded classes, described as a combination of contact and distance calls.

Distance calls overlap least with all other classes in temporal features and are consistently structurally distinct. This call type is the only one in our dataset that is typically used in the absence of visual contact. It has been hypothesised that graded signals typically evolve in species with visual contact^32^, where other nonacoustic cues may disambiguate function. This hypothesis provides a possible explanation for the distinctness of distance calls: Being able to identify the caller and context from their vocalisation is more important when visual contact is absent or limited.

The three to four broad acoustic groupings described above were generally clustered accordingly by all three algorithms. Leiden tended to subdivide recruitment, triumph and alarm call groupings, similar to the human labels, especially with larger dataset sizes (see Fig. S14). Cases with more clusters typically reflected subdivisions within these broader regions rather than entirely separate call types, highlighting both the graded structure of the repertoire and algorithm-specific sensitivity.

#### Number of call classes

The embedding of the data typically resulted in three to four visually largely separate groups when using representations other than LFCCs, consisting primarily of samples from the following classes: (1) ‘contact’, (2) ‘recruitment’ and ‘triumph’, (3) ‘departure’ and ‘alarm’, and (4) ‘distance’. Groups one and two were only distinct when embedding audio feature vectors. The structural overlap of triumph/recruitment calls, as well as alarm/departure calls, suggests that these call types can only be clearly distinguished when taking other information like the behavioural context, rhythm or sequential context into account. Nonetheless, the human-defined number of call types is in line with the average number of classes suggested by unsupervised methods.

### Limitations

This study provides insight into the vocal repertoire of the adult greylag goose, but does not include all of the previously described call types. In particular, hisses were excluded due to insufficient data, and call types mentioned in the early literature like the greeting or locomotion call^25^ were not included in the data collection process. Lorenz describes both of these call types as higher intensity contact calls, otherwise only distinguishing them by context and posture. Based on personal observations, we suspect these calls to be contextual variants of the contact call. Thus, our analysis should not be mistaken for an investigation into the size of the greylag goose vocal repertoire, but rather an exploration of its structure.

Additionally, the analysed recordings were collected in a field setting and contained some background noise even after applying a noise reduction algorithm. Furthermore, distance to the vocalising individuals differed between recordings and therefore amplitude was normalised but could play a useful role in subsequent analyses. This discards information on relative loudness of call types, but could also leave structural artefacts in the energy distribution of the call, specifically less energy remaining in the higher frequency range for more distant calls, which may lead to distances in UMAP projections that do not accurately reflect the structural differences of the emitted sounds.

Furthermore, we did not investigate the rhythmic and sequential domain of the vocalisations, analysing only single syllables. As the call types have been consistently described in terms including their rhythm and syllable count, this remains a considerable limitation of our study. However, as we already find the individual syllables to match human-labelled classes to a considerable degree, further investigating sequencing and rhythm may enable us to distinguish acoustically overlapping call types like triumph or alarm calls.

Leiden community detection has, to our knowledge, not yet been used in bioacoustics analyses, and was found to produce results comparable to those of more commonly used clustering algorithms. It is important to note that HDBSCAN and k-means clusterings based on 2, 20 and 100-dimensional UMAP embeddings were compared to Leiden clusterings operating on the nearest-neighbours graph directly, which potentially favors Leiden since additionally reducing dimensions might already exclude relevant information. On the other hand, Leiden has been described as generating too many clusters^33^. This should be considered in future work, even though the average number of predicted clusters matched the number of human labels best when using this algorithm. Additionally, we only applied one partitioning approach for the Leiden clusterings, and the nearest neighbours graph was not preprocessed before detecting clusters, providing an opportunity for future algorithmic improvements.

Lastly, we emphasize that this study does not aim to analyse the performance of different clustering algorithms beyond the scope of comparing them to human classifications (based on behavioural context, in a well-studied species). Our methods should be extended using datasets of different species and in-depth analyses before drawing strong conclusions about the methods regarding other species. We strongly recommend that a vocal repertoire analysis always be combined with extensive behavioural observations, and suspect that no single algorithm or representation type fits all applications and species.

## Conclusion

The greylag goose has been a model system in ethology since the field’s early days, but the species’ vocal repertoire has not previously been quantitatively investigated. We used different data representations and clustering algorithms to investigate the greylag goose repertoire, building on years of behavioural observation of individually marked birds. We found both graded and distinct signals, largely congruent with human-labelled signal classes. However, our results show that the choice of the data representation and the algorithm used for investigating a species’ vocal repertoire can strongly influence the results obtained, even for this well-studied species, where some “ground truth” knowledge is available. This indicates that, when investigating vocal repertoires in less-understood species using unsupervised ML algorithms, considerable caution is warranted. We found that audio feature vectors allowed a more detailed visualization of the acoustic space than the other, spectrogram-based data representation types analysed here, and led to clusterings most congruent with human labels. All of the three tested clustering algorithms produced good results overall, but there was a high variation in the number of predicted classes and the congruence with human labels, depending on the choice of the data representation type as well as the dataset size and balance. When investigating vocal repertoires, it is thus important to compare different types of data representation, and carefully consider the algorithms used, especially when estimating the number of signal classes. Nonetheless, our analyses illustrate the power of modern machine learning techniques to illuminate the structure of a vocal repertoire, even for a well-studied species, and demonstrate the promise of applying these analyses to species with less-studied vocal repertoires.

## Materials and methods

### Study system and site

Our study population is a free-flying flock of greylag geese (*Anser anser*) at the Konrad Lorenz Research Center in Grünau im Almtal, Austria. The flock was introduced to Grünau im Almtal in 1973 by Konrad Lorenz with long-term research continuously ongoing since then, including individually color-banded and marked birds, and collection of behavioural and life history data, with regularly updated data on individual behavioural differences, social networks, and life histories^34–36^.

The flock is non-migratory, habituated to humans, and food-supplemented twice a day at 8 am and between 4 pm and 7 pm at Auingerhof, Grünau im Almtal (47°48’49.7412” N, 13°56’51.72” E). After the morning feeding, individuals fly in groups to the nearby Cumberland Gamepark (47°48’37.6704” N, 13°56’53.9196” E) where they may receive food from visitors of the park. After the afternoon feeding, they move to Lake Alm (47° 45’ 12.1356’’ N, 13° 57’ 24.9948’’ E), Oberganslbach (47° 47’ 36.762’’ N, 13° 56’ 57.2316’’ E), or the Gamepark to sleep. Data collection took place September 2020 to September 2025. Table S4 summarises information about the flock.

### Data collection

The Konrad Lorenz Research Centre has gathered an archive of audio recordings containing information on the identity of the calling individual and the attributed call type since 2020. The original dataset collected from this population contained 11,015 WAV files labelled with caller identity, call type, location and time, specifications concerning the recording gear, and sometimes behavioural context. The recordings were collected between September 2020 and November 2023 at Auingerhof, Oberganslbach and the Cumberland Gamepark. Sampling rates ranged between 41.1 and 48 kHZ at 16-bit-integer to 32-bit-float quantisation. Because the original dataset had strongly differing sample sizes for the different call types, it was expanded for this study with targeted audio recordings of alarm and triumph calls. This additional data collection took place in March, August and September 2024, and March 2025 at the locations described above (also see Figs. S15–S17). Vocalisations were recorded in an opportunistic manner at a distance between 2 and 5 m from the vocaliser using a Sennheiser MKE 600 directional microphone (Sennheiser electronic SE & Co. KG, Germany) attached to a Zoom F3 (32-bit-float quantisation at 48 kHZ) or H5 field recorder (24-bit-integer quantisation at 48 kHz) (Zoom Corporation, Japan).

This study complies with all current Austrian laws and regulations and was supported by Animal Experiment License Number 66.006/0026-WF/V/3b/2014 issued by the Austrian Federal Ministry for Science and Research (EU Standard, equivalent to the Animal Ethics Board). All data collected for this study were obtained using minimally invasive recording and observation methods that did not involve any animal handling. Birds were free-moving and could move away or fly away at any time.

### Segmentation

All recordings were subdivided by hand into smaller audio tracks containing a single call type with one or more utterances using Audacity version 3.4.0 (Audacity Team, 2023). The tracks in the database were downsampled to 44.1 kHz to achieve consistent sampling rates, and quantisation was set to 16-bit. Subsequently, the noise of all sound files was reduced through a spectral gating approach using the Python package *‘*noisereduce’^37^ after applying a 0.2 to 16 kHz bandpass filter (SciPy^38^, version 1.14.1). Since the original segmentation was performed by several researchers with different preprocessing of call segments, new clips were extracted from the noise-reduced original tracks. To make re-segmenting of more than 11,000 calls feasible, this was done semi-automatically, using spectrogram cross-correlation to detect the original segment boundaries in the original track and exporting detected timestamps with the original call type label from the database. All call type annotations as well as segmentations were then manually verified in Raven Lite^39^ version 2.0.5. This approach ensured consistent segment boundaries, preprocessing, as well as call categorisation. All segments containing overlapping vocalisations, high levels of remaining noise, or unclear call type labels were excluded (*n* = 4379), leading to a remaining 12 hours of tracks in total. Further processing was accomplished using Python (version 3.11.10).

### Preprocessing

**Fig. 7:**
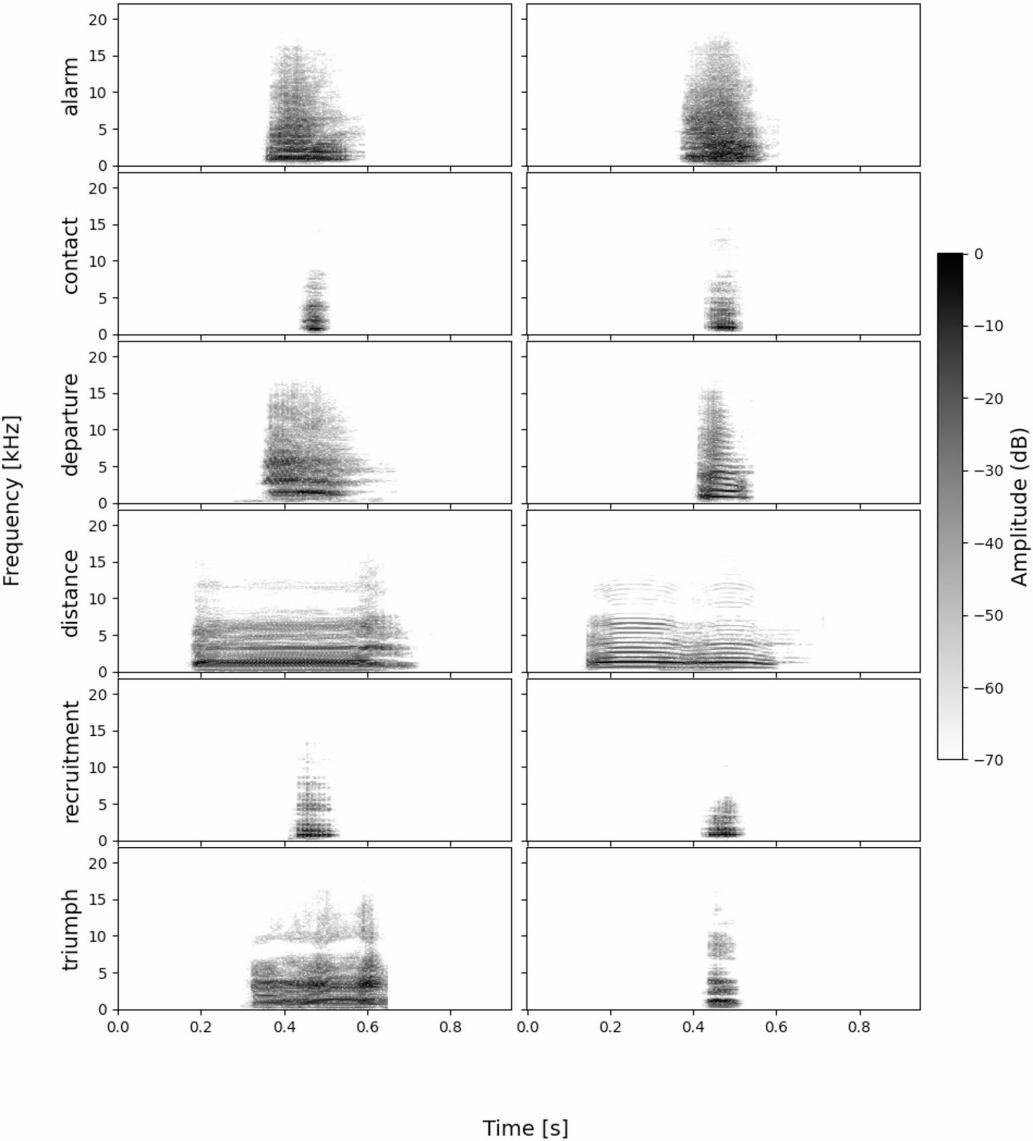
Spectrograms of peak-normalized example calls for the different call type classes. Alarm and departure as well as contact and recruitment calls are structurally very similar. Triumph calls vary in length substantially. Tonality varied internally in some of the classes (e.g. see triumph call examples).

Each segment was individually peak-normalised to prevent the influence of varying recording distances, although call types may have consistently different loudness levels among them. Then, leading and trailing silences were cut using a threshold of 35 dB SPL down with the *trim* function in the Python package ‘librosa’^40^ (version 0.10.2) to ensure consistent segmentation. The final dataset contained single syllables of 6651 calls in total, with 228 human-labelled as alarm, 1393 contact, 958 departure, 486 distance, 3214 recruitment, and 238 triumph calls of durations between 0.039 and 0.99 seconds. Figure 7 shows example spectrograms of these different call types. This imbalance in number of calls results from some call types being uttered much more frequently, but is also an artefact of data collection targeting specific call types.

### Feature extraction

The data were represented in four ways: (1) vectors of 23 audio features (see below), (2) 16 linear frequency cepstral coefficients (LFCC), (3) Fourier transformed linear frequency spectrograms, and (4) latent vectors of length 128 from a variational autoencoder trained on spectrograms^7^. For the spectrogram-based representation types, the segments were zero-padded logarithmically to the same length of logarithmically scaled durations to account for the large difference in call lengths. For this, the duration of the call segments was log-rescaled and then zero-padded from both sides to the length of the longest call segment.

#### Audio feature vectors

From the audio segments, eight temporal, nine spectral, and six spectro-temporal features were extracted using the Python packages ‘numpy’^41^ and ‘SciPy’^38^ (version 2.3.2 and 1.14.1). All extracted features, details on their calculation and the specific python packages used are listed in Table S5.

#### Linear-frequency cepstral coefficients (LFCCs)

LFCCs were calculated using custom python code with 97% preemphasis, a Hann window of size 512 (12 ms) with a 50% overlap and a linear filter bank of 32 triangular filters. The calculation of LFCCs quantifies the gross shape of the spectrum, and is well-suited for formant analysis. However, its performance on data with a low signal-to-noise ratio has proven to be disadvantageous^42^, and considerable fine spectral structure is discarded through the calculation.

#### Spectrograms

Linear frequency scale spectrograms were computed using *scipy.signal.stft* with a Hann window of size 512 samples and an overlap of 90% and clipped to a dynamic range of − 70 dB SPL. These spectrograms were transformed to two-dimensional arrays with min-max-scaled pixel values between zero and one. Because little energy was left in the upper 40% of rows, these were cut out, resulting in an array size of 25 × 201. All of the above data representations were scaled using the class ‘StandardScaler’ of the preprocessing module in the ‘scikit-learn’^43^ Python package (version 1.5.2) before further analysis.

#### Variational autoencoder (VAE)

Finally, these spectrograms were used to train a VAE with a latent vector of size 128. The VAE was constructed using the package ‘PyTorch’^44^ (version 2.4) with a decoder of five convolutional and two fully connected layers (see Figure S18 for detailed description). The weights were Glorot initialized (*torch.nn.init.xavier_uniform_*, ‘TorchVision’^45^ version 0.17.1) and the model trained using an Adam optimizer with a learning rate of 1e^− 6^ to reduce overfitting. All classes were randomly sampled up to 500 times and a random roll applied to the spectrograms on the time axis for training using *torch.roll* of the ‘TorchVision’^45^ package (version 0.17.1). To simplify the layer design, spectrograms were resized to 24 × 128 pixels using the ‘TorchVision’ *transforms.Resize* class. To ensure the possibility of future data generation while weighting a more detailed representation higher than a continuous latent space for clustering, we used the sum of the full mean squared error plus 10% of the Kullback-Leibler divergence as the loss function (custom python code).

### Feature embedding

For the clustering pipeline, we employed the entire dataset, along with random subsets of sizes up to 50, 100, 200, and 500 per human-labelled category for every representation type. Due to insufficient sample size, the categories ‘alarm’ and ‘triumph’ were discarded for the subset size 500. These datasets were randomly drawn and analysed 20 times for each subset size to estimate random error. In addition to the randomly drawn subset sizes, the pipeline was bootstrapped five times with the entire dataset, due to computational time constraints.

The above described treatment was repeated for dimensionalities of 2, 20 and 100 as follows: From the subsamples, nearest neighbour graphs were extracted using UMAP based on Euclidean distance using the Python package ‘umap’^46^ (version 0.5.6). The argument *n_neighbors* was set to 20% of the subset size or 100 when using the entire dataset. The same package was used to embed the resulting graphs into the three different dimensionalities (2, 20, 100) with the argument *min_dist* = 0, as advised for clustering^47^.

### UMAP

UMAP is a feature extraction approach that maps the structure of the original data into a lower dimensional latent space, combining the original features of the data in a nonlinear fashion. Each resulting dimension in the new latent space represents a feature. This allows preserving much of the structure and variance of the original dataset without the need for prior assumptions about the character of the relationships within the data[9] .

Subsequently, three clustering algorithms were applied: k-means^48^, HDBSCAN^49^, and Leiden community detection (hereafter: ‘Leiden’)^50^. Leiden was applied to the nearest neighbours graph directly, whereas k-means and HDBSCAN were used to cluster the two-dimensional UMAP projections. The resulting projections were compared to random distributions using the Hopkins statistic as described in Hopkins and Skellam^51^ with a subset size of 7% (custom code, Python 3.11.10).

#### k-means

The k-means algorithm^48^ generates a given number *k* of clusters by assigning data points to clusters using the mean of each cluster in an iterative manner. k-means was implemented using the Python package ‘scikit-learn’^43^ (version 1.5.2). The number of clusters was varied, and finally set to obtain the highest silhouette score, which measures how well the clustering fits the projected datapoints, while keeping the default values for all other parameters.

#### HDBSCAN

HDBSCAN^49^ is a hierarchical density-based clustering algorithm, based on DBSCAN. The DBSCAN algorithm allows assigning data points to an unidentified number of possibly non-spherical clusters, by calculating the number of neighbouring points in a defined radius *epsilon* and assigning data points above a chosen threshold count of neighbours to a cluster. In HDBSCAN, the radius is varied and the most stable clusters are chosen as the final clusters. We used the Python package ‘hdbscan’^49^ (version 0.8.39) to implement the algorithm, setting *min_cluster_size* to 1% of the dataset, leaving the threshold *cluster_selection_epsilon* at 0 to receive the original HDBSCAN results, and optimising the parameter *min_samples* to maintain the highest silhouette score^52^ (scikit-learn.metrics^43^, version 1.5.2).

#### Leiden community detection

Leiden^50^ finds partitions of a graph, optimising its modularity. Modularity compares the number of in-community edges in the graph to the expected value of in-community edges in the same community division with random connections^53^. Leiden was applied using the Python package ‘leidenalg’^50^ (version 0.10.2) with partition type *ModularityVertexPartition*. The nearest neighbours graph obtained from UMAP was transformed using ‘igraph’^54^ (version 0.10.15).

#### Statistical analysis

All resulting clusters were compared to human labels using V-measure^55^(scikit-learn.metrics^43^, version 1.5.2), setting $\:\beta\:=1$ to weigh homogeneity and completeness equally, as well as adjusted Rand score using the same Python package. We employed a Gaussian generalized linear mixed model with an identity link function to analyze the influence of representation types, algorithms, balance and size of the dataset, and dimensionality on V-measure (*V-measure ~ Representation + Algorithm + Balance + Dataset Size.z + Dimensionality.z*) using the R (^56,version 4.3.3^) package *glmmTMB*^57^.

### Classification

**Fig. 8:**
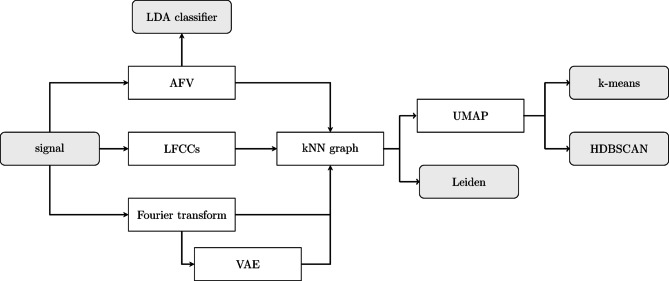
Analysis pipelines for the audio data: Raw data were transformed into linear frequency spectrograms, linear frequency cepstral coefficients and audio feature vectors. Additionally, spectrograms were used to train a variational autoencoder. Of all representations, a nearest neighbours graph (kNN graph) was extracted and either used to embed the data into 2, 20 and 100 dimensions using UMAP, or fed into Leiden community detection. All resulting embeddings were then clustered using k-means and HDBSCAN. This pipeline was applied to random subsamples of the original dataset with class sizes of 50, 100, 200 and 500 (resampled 20 times each). Graphs were computed based on Euclidean distances. Alarm and triumph calls were excluded at a maximum subset size of 500 due to insufficient sample sizes.

To gain more detailed insight into the acoustic characteristics of the call types as well as to check the validity of the class distinctions, we trained a Linear Discriminant Analysis (LDA)^58^ classifier on the audio feature vectors using ‘scikit-learn’ with a least-squares solver, leaving all other parameters at their default values. LDA maximises the ratio of between-class to within-class variance by identifying linear combinations of features that separate the given classes. The model was trained on a subset of the original dataset with class sizes capped at 500 of which 20% were set aside for testing. The f1 score, a common measure for predictive performance, was used for evaluation and tested against chance using a permutation test with 500 permutations and five-fold stratified cross validation with non-overlapping groups. Figure 8 provides a comprehensive overview of the analysis pipeline.

## Supplementary Information

Below is the link to the electronic supplementary material.

Supplementary Material 1

## Data Availability

The dataset analysed during the current study is openly accessible at https://phaidra.univie.ac.at/detail/o:2180221.

## Code availability

The code used to retrieve the data representations is openly accessible at: github.com/lgies23/biosonic and https://pypi.org/project/biosonic/. The full code to reproduce the analysis pipeline of this article is openly accessible at: https://gitlab.phaidra.org/lenag23/grgoovoc25.
