# Supplementary material for: Investigating the structure of the greylag goose vocal repertoire: what can unsupervised methods tell us?
Source: Sci Rep. 2026 Jun 3;16:25219. doi: 10.1038/s41598-026-51404-2 (PMC13470216; doi:10.1038/s41598-026-51404-2)
Supplement: Supplementary file 1 [file 41598_2026_51404_MOESM1_ESM.pdf]

Supplementary material

Table S1 Average number and range of predicted groups per clustering algorithm averaged over all tested dimensionalities.

Algorithm	Number of clusters (range)	Mean ± SD
kMeans	2 - 16	4.25 ± 2.58
HDBSCAN	2 - 33	6.14 ± 6.40
Leiden	3 - 11	5.38 ± 1.95

Table S2 Overlap between hand-labelled and automatically clustered classes averaged over all tested dimensionalities for the different data representation types and algorithms.

Representation	Mean V-measure ± SD	Mean adj. RS ± SD	Algorithm	V-measure ± SD	Adj. RS ± SD
Audio feature vectors	.556 ± .108	.469 ± .151	kMeans	.566 ± .108	.482 ± .155
			HDBSCAN	.556 ± .148	.461 ± .202
			Leiden	.547 ± .035	.463 ± .056
LFCC	.437 ± .065	.268 ± .083	kMeans	.476 ± .068	.317 ± .070
			HDBSCAN	.381 ± .047	.188 ± .070
			Leiden	.453 ± .028	.300 ± .035
Spectrograms	.548 ± .059	.399 ± .068	kMeans	.561 ± .038	.413 ± .050
			HDBSCAN	.563 ± .079	.405 ± .093
			Leiden	.520 ± .039	.381 ± .049
VEA embeddings	.532 ± .048	.397 ± .061	kMeans	.541 ± .029	.410 ± .040
			HDBSCAN	.523 ± .070	.380 ± .083
			Leiden	.532 ± .034	.399 ± .047

Table S3 Summary of the statistical model.

V-measure					
Predictors	*Estimate*	*95% CI*	*p*	$\chi 2$	*Df*
Intercept	0.38	0.36 – 0.40	**<0.001**		
Representation (LFCC)[1]	-0.12	-0.13 – -0.11	**<0.001**	1545.7753	3
Representation (Spectrogram)[1]	-0.01	-0.02 – -0.00			
Representation (VAE)[1]	-0.02	-0.03 – -0.02			
Algorithm (k-means)[2]	0.03	0.02 – 0.04	**<0.001**	112.6996	2
Algorithm (Leiden)[2]	0.01	0.00 – 0.01			
Dataset size (z)	0.05	0.04 – 0.05	**<0.001**	460.1522	1
Balanced dataset (true)	0.17	0.15 – 0.19	**<0.001**	318.1904	1
Dimensionality (z)	-0.00	-0.00 – 0.00	0.637	0.2225	1
Observations	3060				
R^2 / R^2 adjusted	0.409 / 0.407				

[1]*reference value: Audio Feature Vectors*

[2]*reference value: HDBSCAN*

Bold values indicate statistical significance of the overall term at alpha set to 0.05.

Table S4 Information about the study population during the years of data collection.

Year	# birds	Avg age ± SD [years]	# females	# males	Paired [%]	# hand reared
2020	114	6,78 ± 5,56	45	66	67,5	14
2021	128	6,24 ± 5,91	47	77	59,4	11
2022	120	6,91 ± 5,71	42	65	62,5	13
2023	92	7,13 ± 5,29	36	55	80,4	9
2024	97	6,08 ± 5,13	36	56	75,3	22

Table S5 Extracted audio features and the corresponding python packages used for calculation.

Domain	Name	Packages Used	Equation
**Temporal**	Duration [s]	`numpy`	
	Temporal quartiles [s]	`numpy`	Time values at the 25th, 50th, and 75th percentiles of the cumulative normalized amplitude envelope
	Temporal centroid [s]	`numpy`	Weighted average of the amplitude envelope
	Temporal std. deviation [s]	`numpy`	Standard deviation of the amplitude envelope
	Temporal skew	`scipy.stats`	`skew()`
	Temporal kurtosis	`scipy.stats`	`kurtosis()`
	Temporal entropy [bits]	`numpy`	Shannon-Wiener Entropy of the normalized temporal amplitude distribution
**Spectral**	Spectral quartiles [kHz]	`numpy`	Frequencies at the 25th, 50th, and 75th percentiles of cumulative spectral energy
	Mean peak frequency [kHz]	`numpy`	Peak frequency of the power spectrum
	Spectral centroid [kHz]	`numpy`	Weighted average of the power spectrum
	Spectral std. deviation [kHz]	`numpy`	Standard deviation of frequencies weighted by power spectrum
	Spectral skew	`scipy.stats`	`skew()`
	Spectral kurtosis	`scipy.stats`	`kurtosis()`
	Spectral flatness	`scipy.stats, numpy`	Geometric mean over arithmetic mean of the power spectrum
	Power spectral entropy [bits]	`numpy`	Shannon-Wiener Entropy of the normalized power spectral distribution
**Spectro-temporal**	Spectrographic entropy	`numpy`	Product of temporal and spectral entropy
	Mean dominant frequency [kHz]	`scipy.signal, numpy`	Dominant frequencies detected using `signal.find_peaks()`
	Min dominant frequency [kHz]	`scipy.signal, numpy`	
	Max dominant frequency [kHz]	`scipy.signal, numpy`	
	Dominant frequency range [kHz]	`scipy.signal, numpy`	
	Dominant frequency modulation [kHz]	`scipy.signal, numpy`	Cumulative difference of the dominant frequency over its range

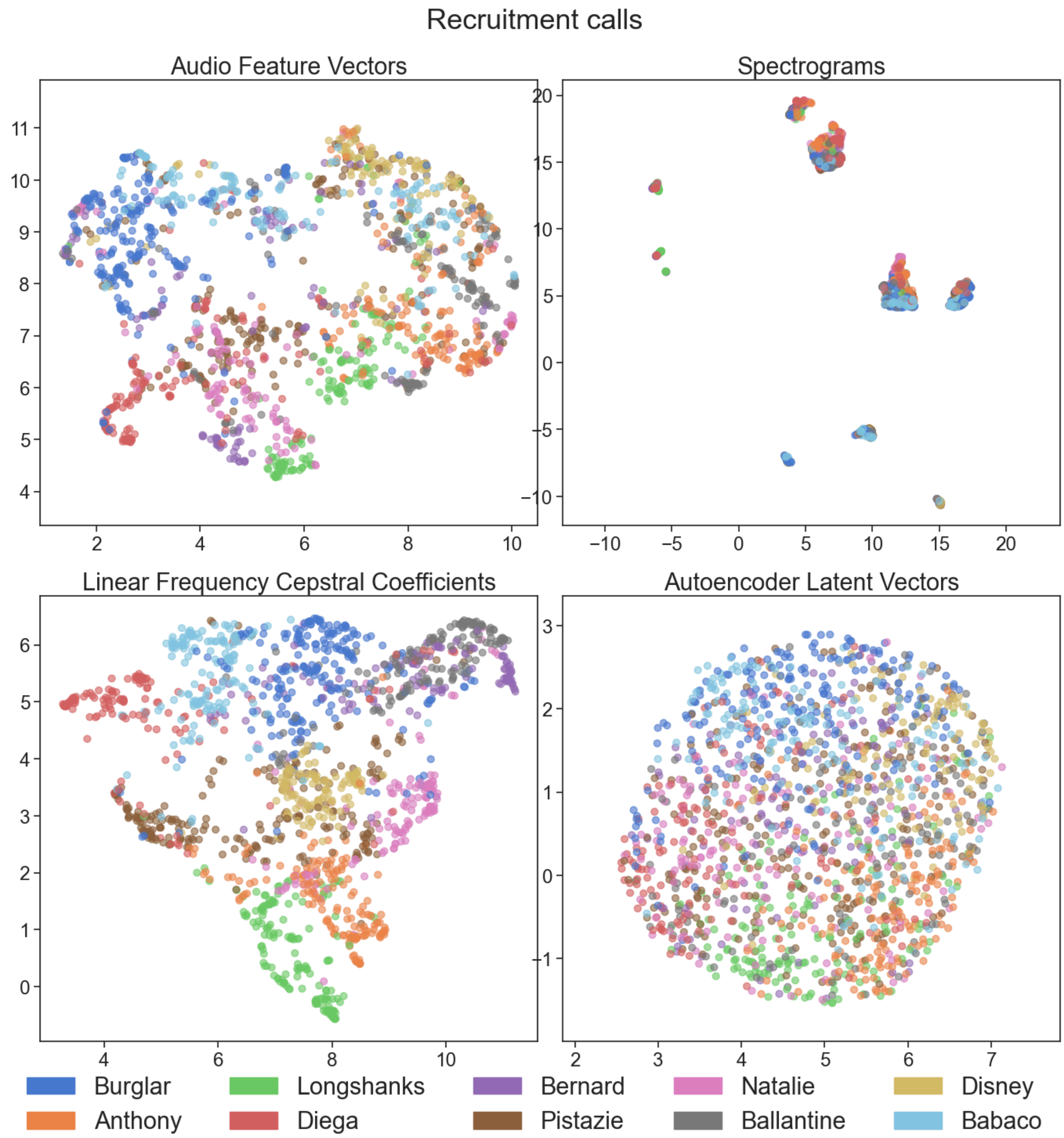

Figure S1 UMAP projections of recruitment calls of the ten geese with the most recorded calls of this type. Colours represent goose ID. Note that the individuals are visually most distinct when embedding LFCCs.

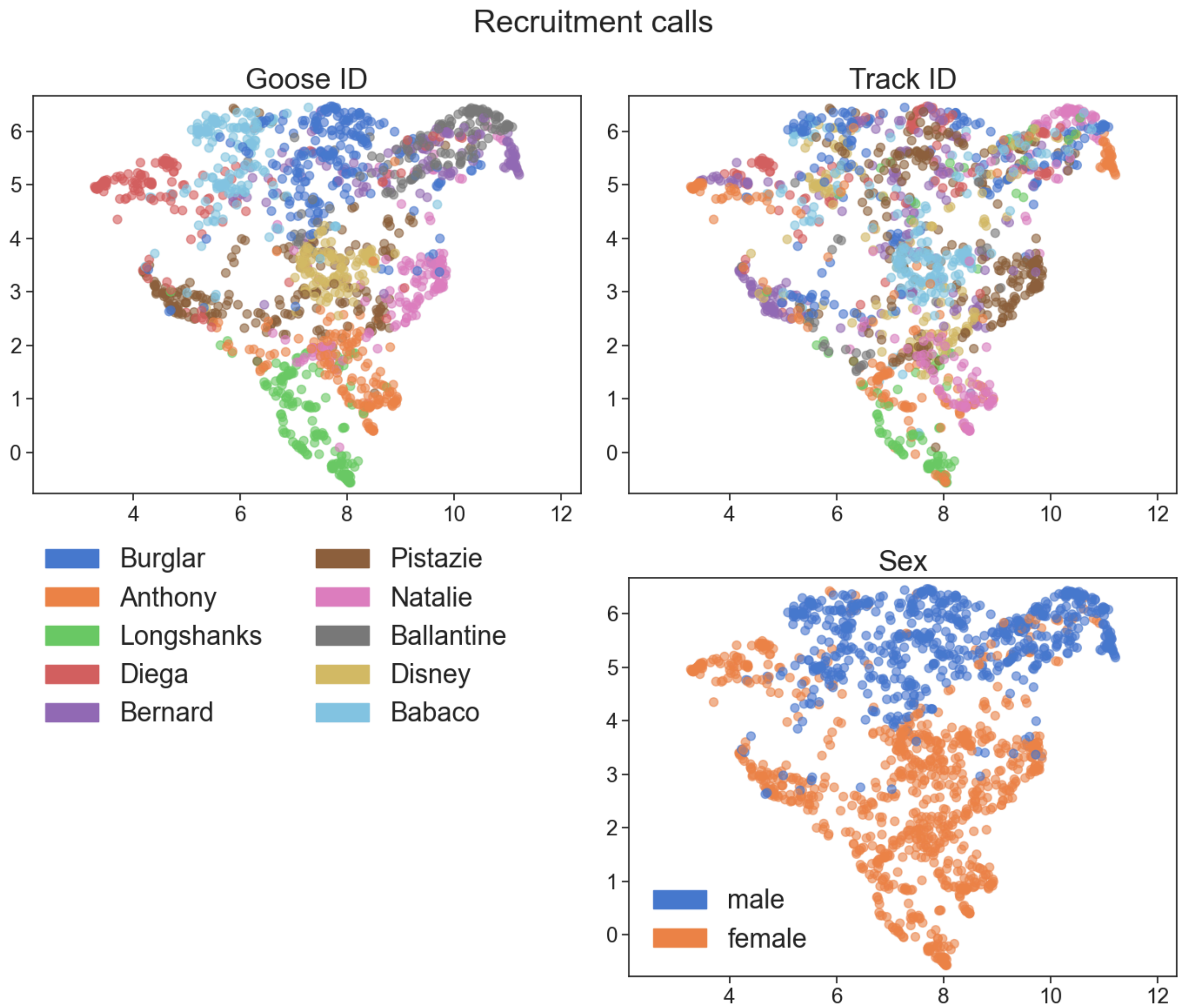

Figure S2 UMAP projections of recruitment calls of the ten geese with the most recorded calls of this type, represented through LFCCs. Colours represent goose ID (top left), track ID (top right) and sex of the vocalizing goose (bottom right).

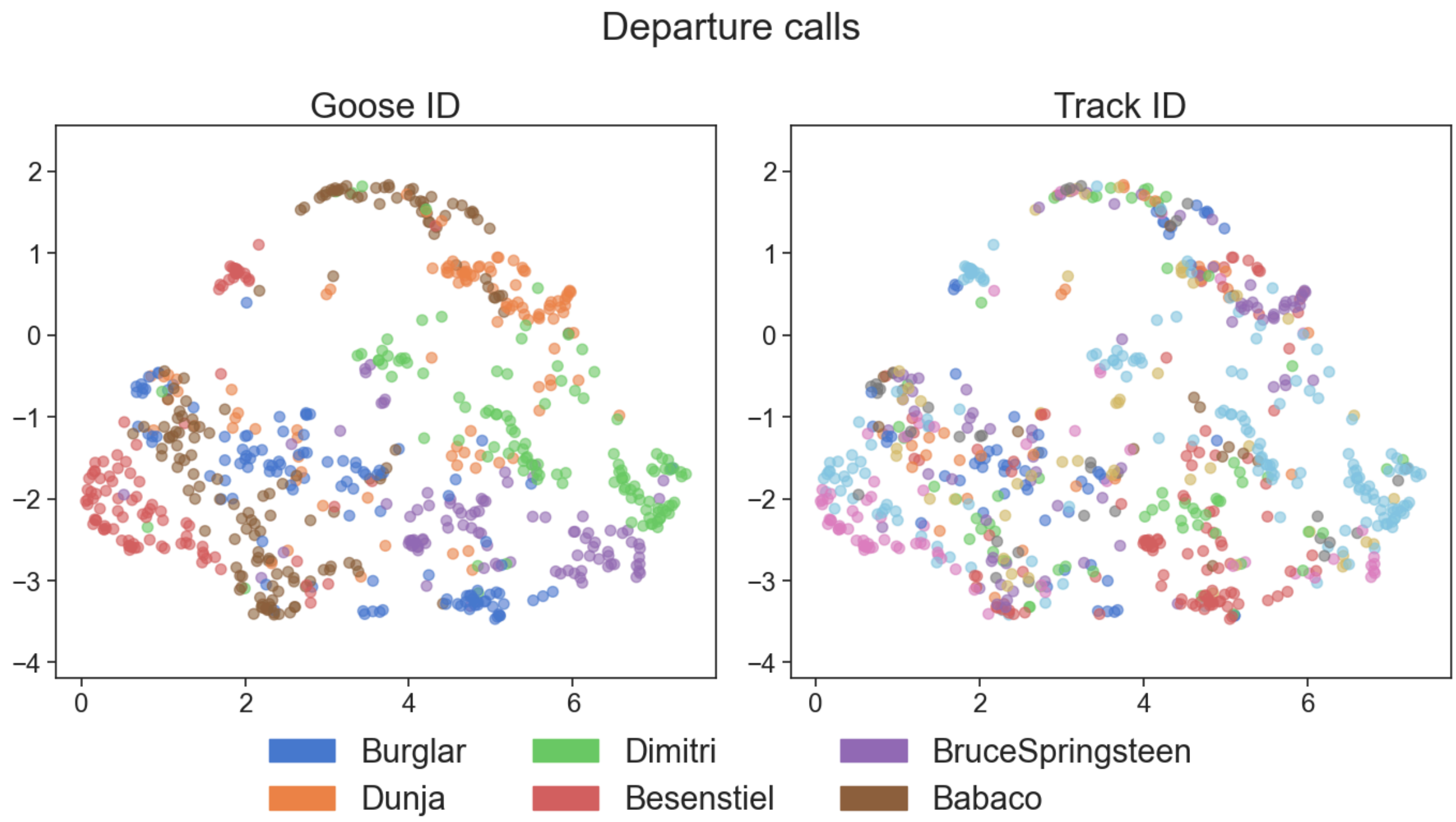

Figure S3 UMAP projections of departure calls of the six geese with the most recorded calls of this type, represented through LFCCs. Colours represent goose ID (left) and track ID (right). Only 3.6 % of recorded departure calls were assigned to a female goose, so all of the data displayed here are associated with male geese.

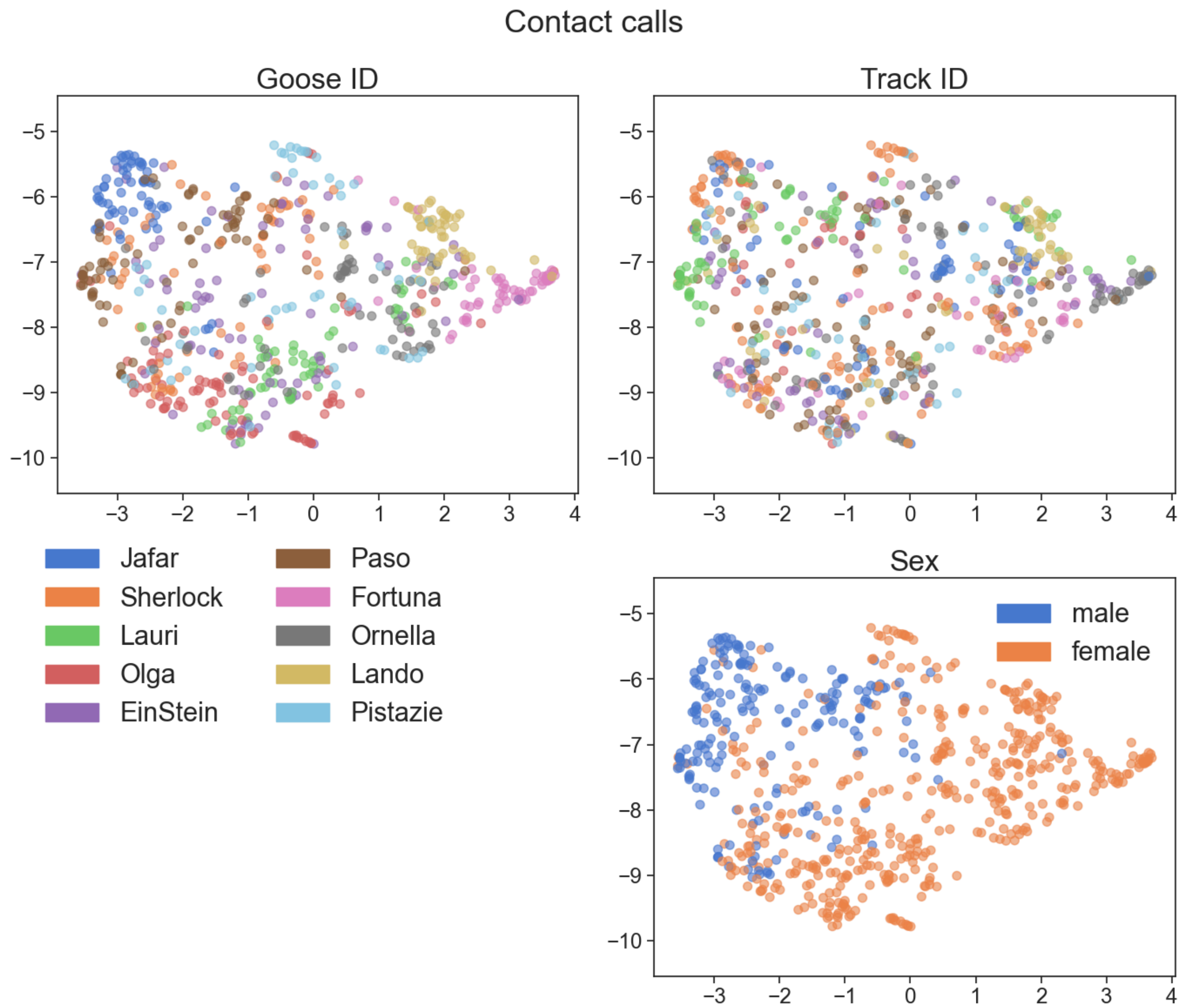

Figure S4 UMAP projections of contact calls of the ten geese with the most recorded calls of this type, represented through LFCCs. Colours represent goose ID (top left), track ID (top right) and sex of the vocalizing goose (bottom right).

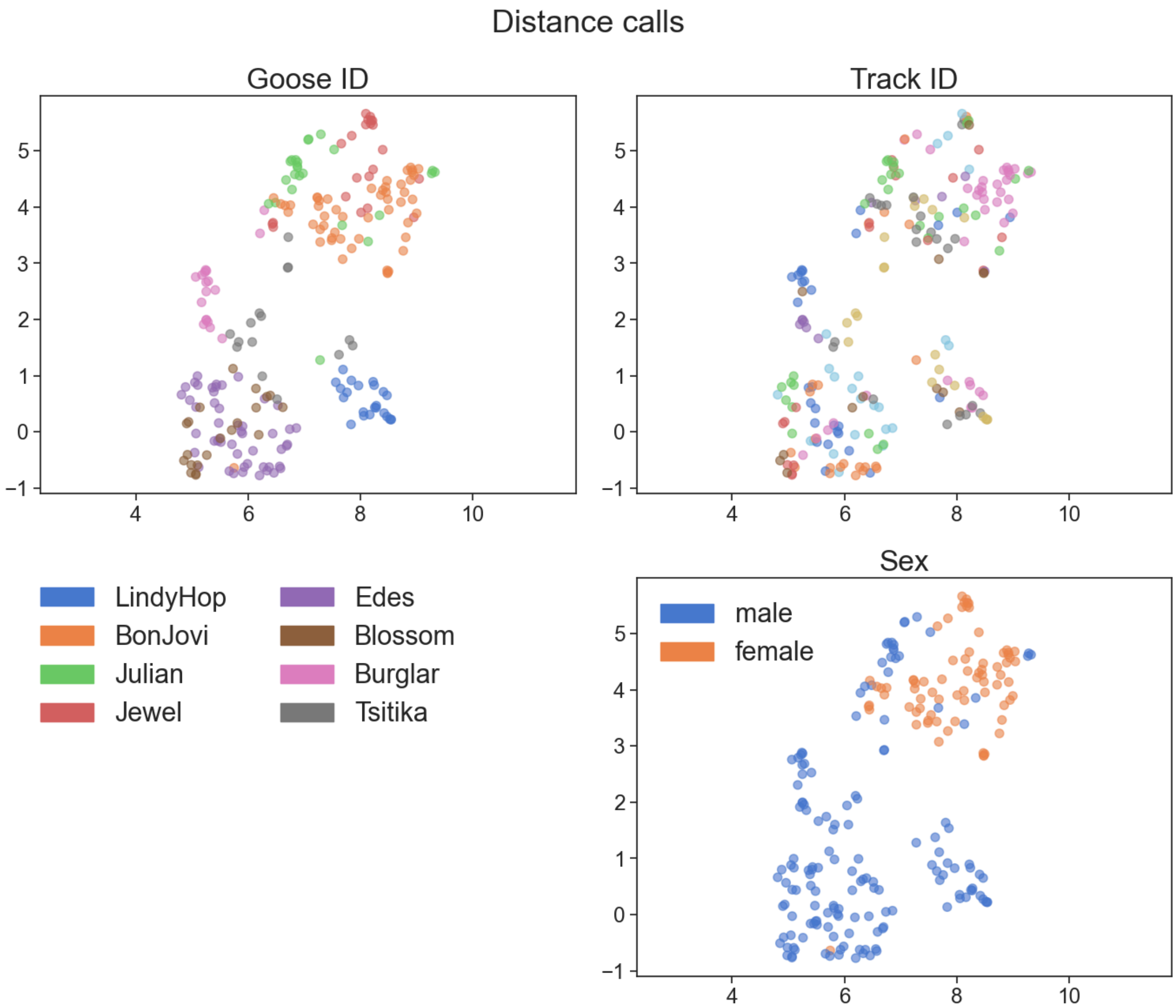

Figure S5 UMAP projections of distance calls of the eight geese with the most recorded calls of this type, represented through LFCCs. Colours represent goose ID (top left), track ID (top right) and sex of the vocalizing goose (bottom right).

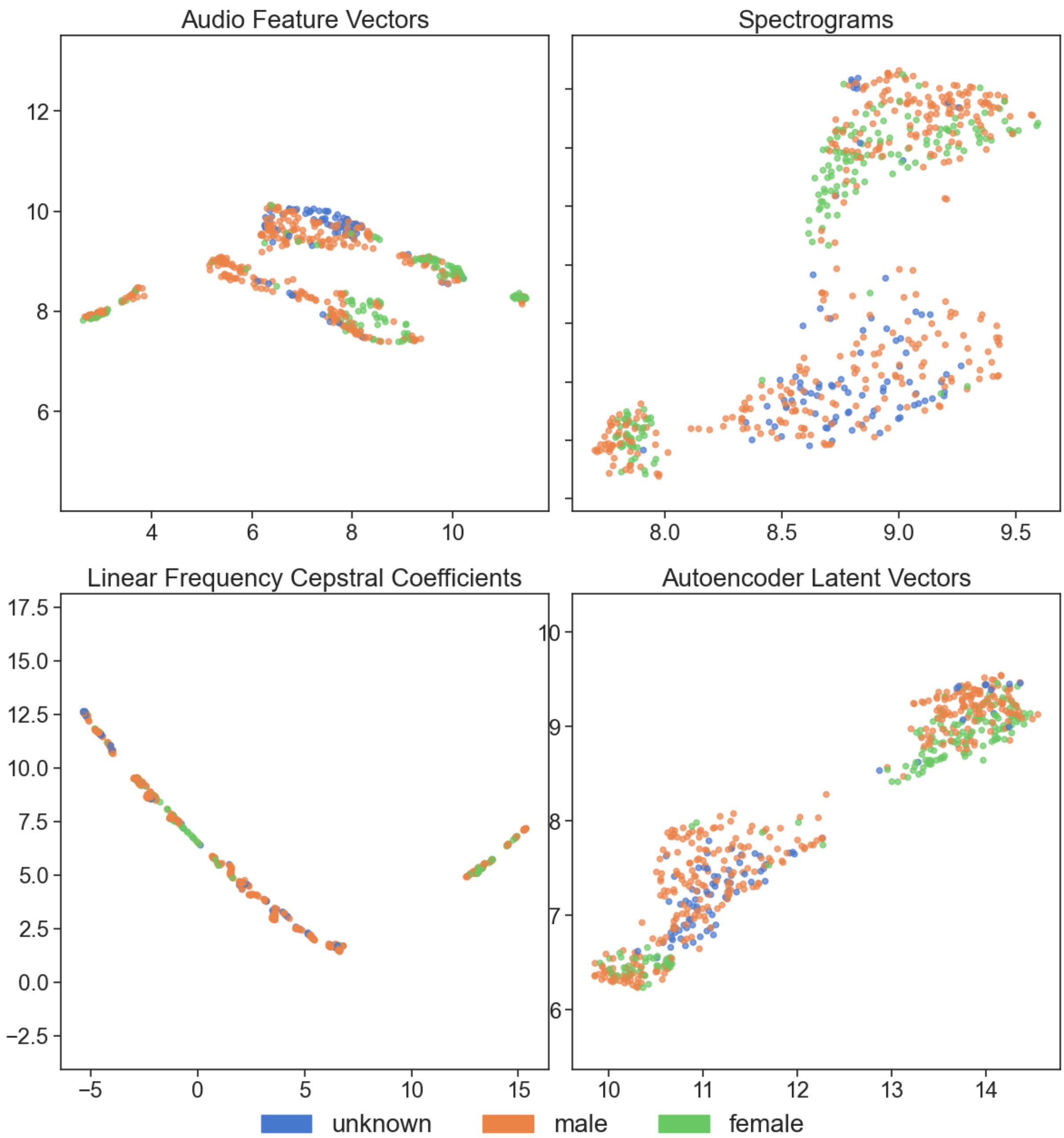

Figure S6 UMAP projections of greylag goose vocalizations color coded by sex of the vocalizing individual. The embeddings are based on a balanced dataset with up to 100 samples per call type category. Also see the following figures S7-11 for more detailed visualizations of the sexual dimorphism in the calls for specific call type classes.

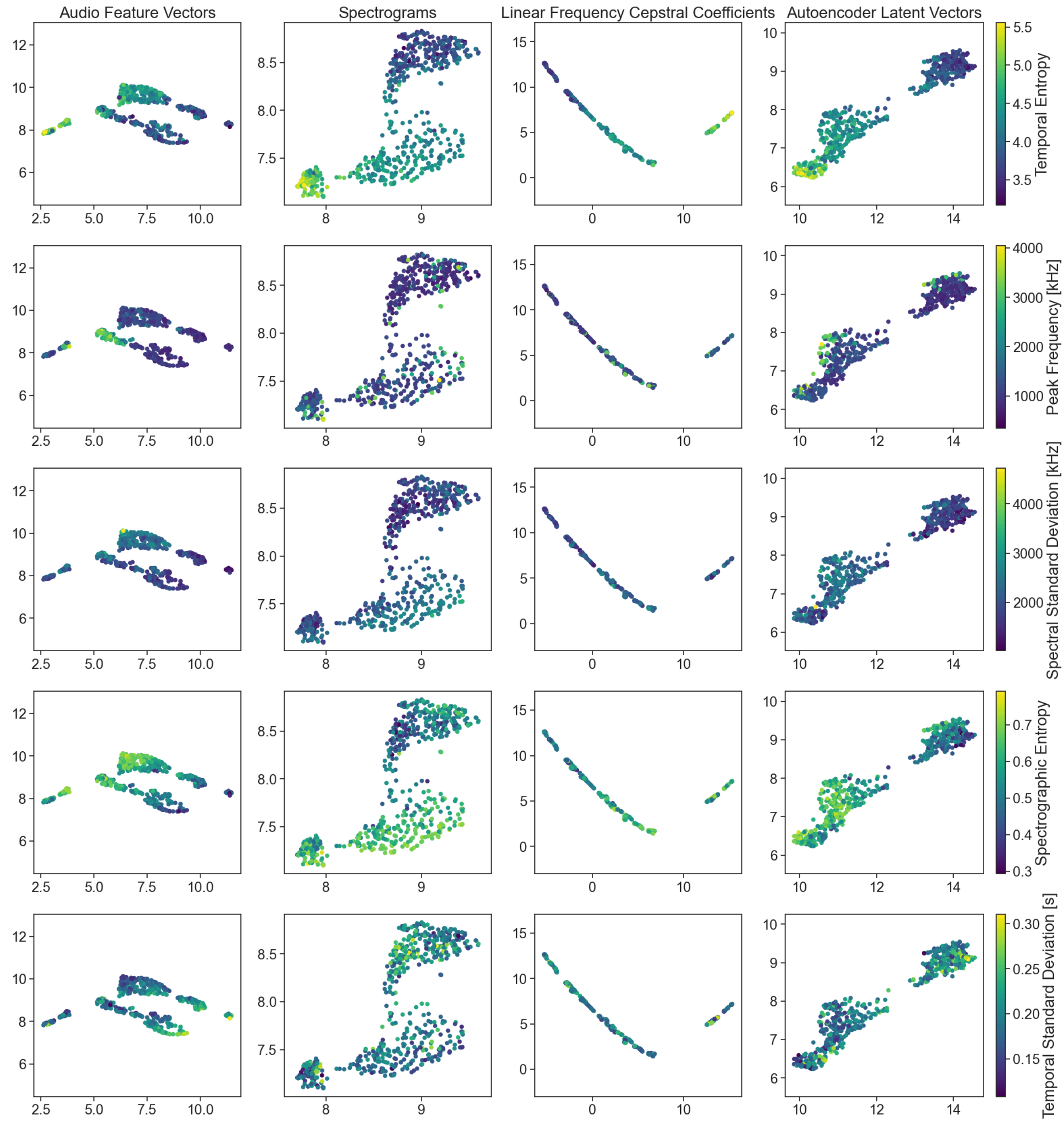

Figure S7 Example projections of every representation, colour-coded for different acoustic features (from top to bottom): normalized temporal entropy, mean frequency in kHz, spectral standard deviation in kHz, spectrographic entropy as the product of temporal and spectral entropy, and temporal standard deviation in seconds.

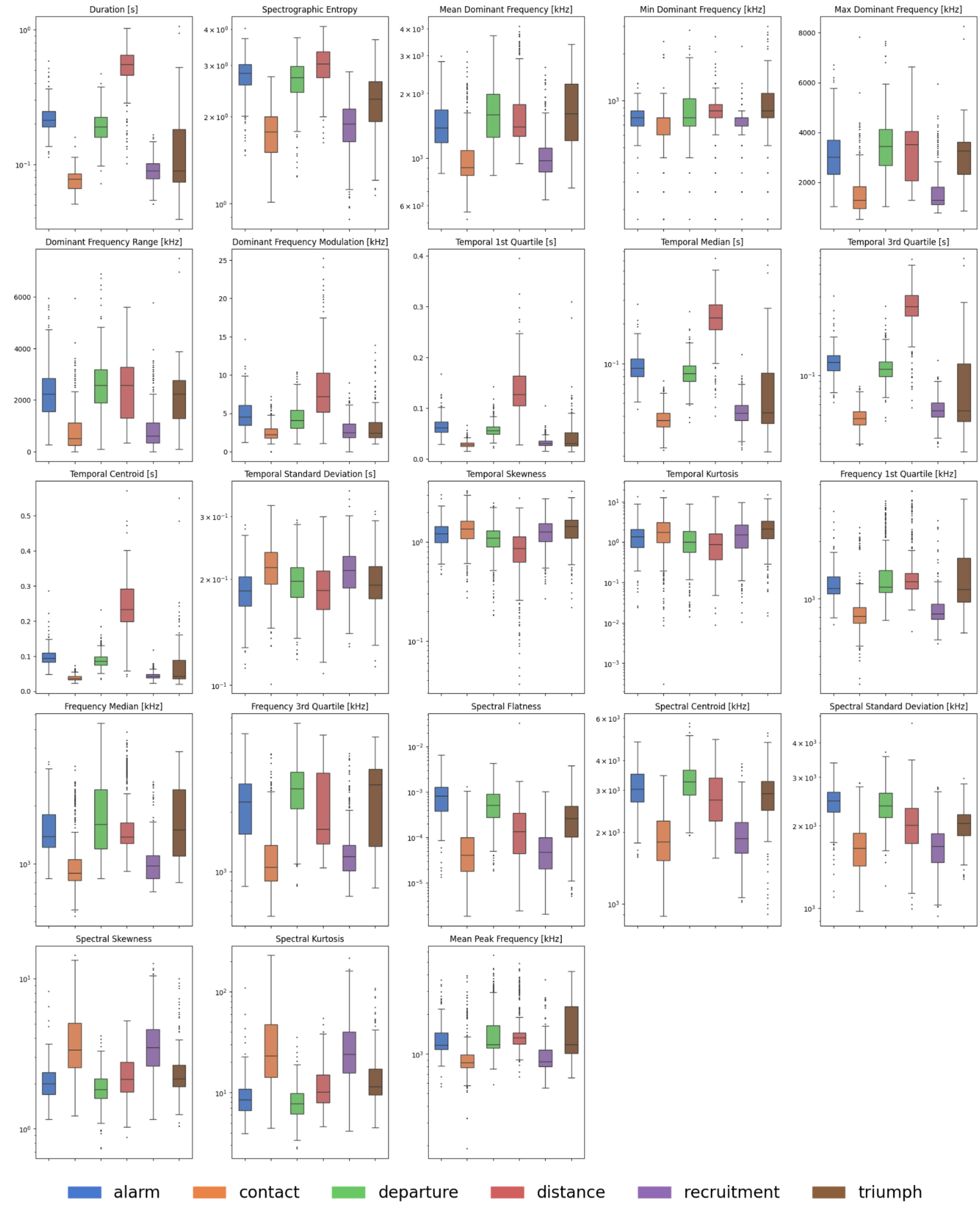

Figure S8 Boxplots for all 23 acoustic features, displaying their distribution per human annotated call type.

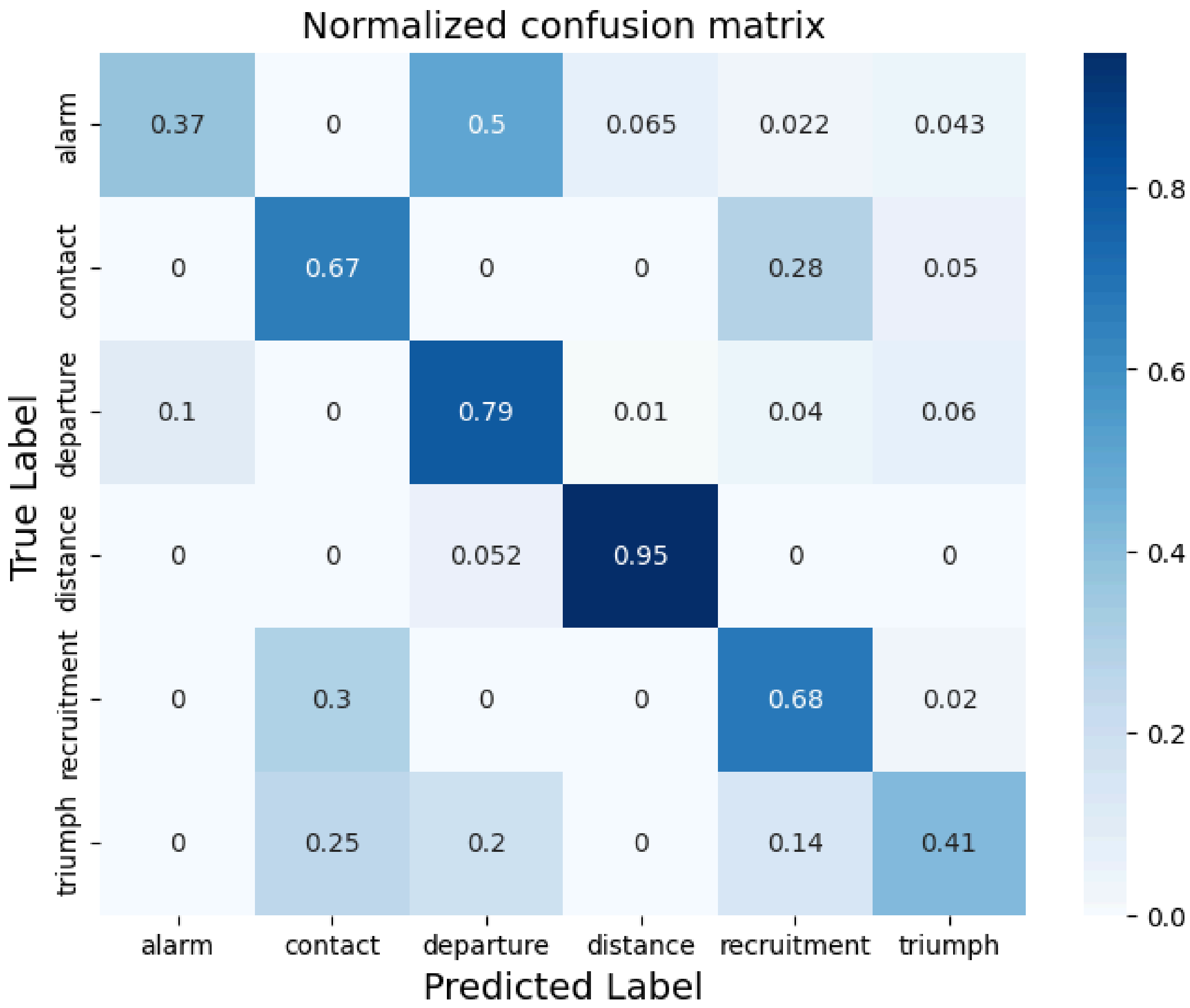

Figure S9 Normalised confusion matrix of the LDA classifier. The model was fitted to a subset of the original dataset with class sizes capped at 500 of which 20 % were set aside for testing. Alarm calls have a low correct prediction rate and are often confused with departure calls. 59 % of the predictions for triumph calls are spread throughout all other categories. Contact and recruitment calls overlap by roughly one third in their predictions respectively. Departure and distance calls were largely classified correctly. The overall accuracy was 60 %, which is above chance (class-wise stratified permutation test, $p = 0.002$).

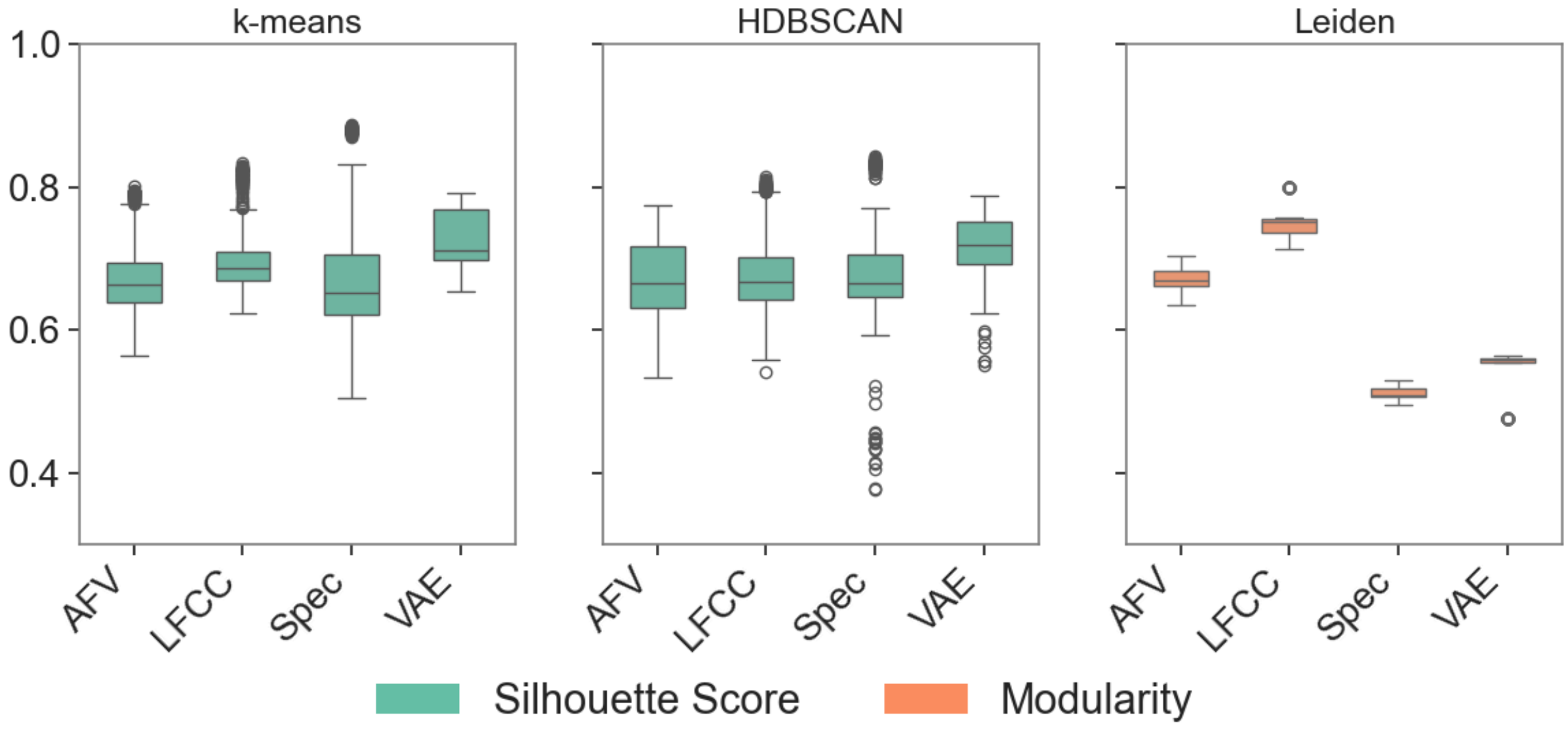

Figure S10 Silhouette scores and modularity for the different algorithms and representation types. Note that the y axis starts at 0.3. Silhouette score ranges from -1 to 1, modularity ranges from 0 to 1.

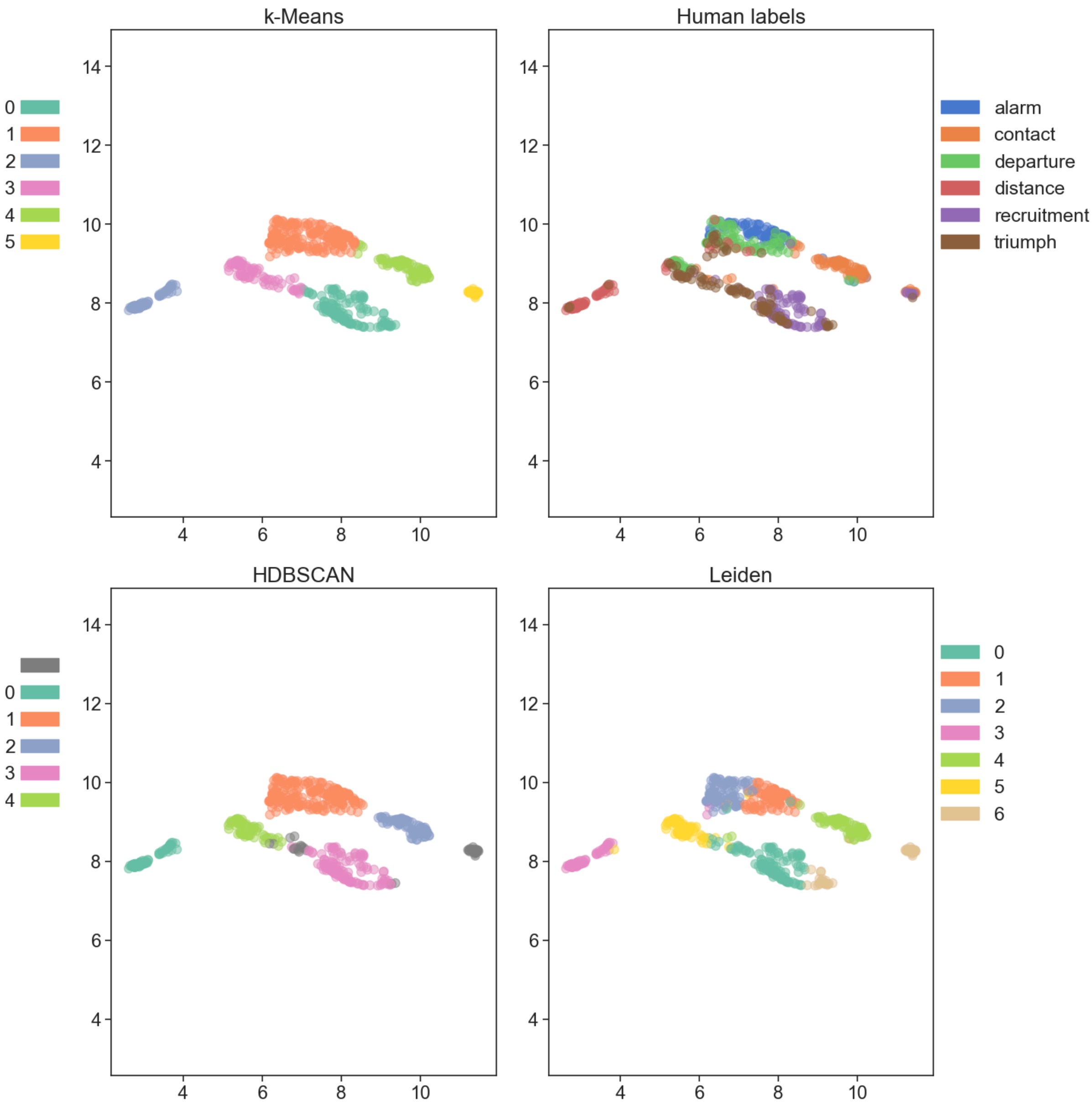

Figure S11 UMAP projections of audio feature vectors. Colours represent human-labelled call types in the upper right plot. Clusters detected using k-means (upper left), HDBSCAN (lower left), and Leiden (lower left) are shown in the remaining plots with random colours for the different clusters. Grey color represents noise for clusters predicted with HDBSCAN. Note, that Leiden does not work on the visualised projections directly but the nearest neighbours graph.

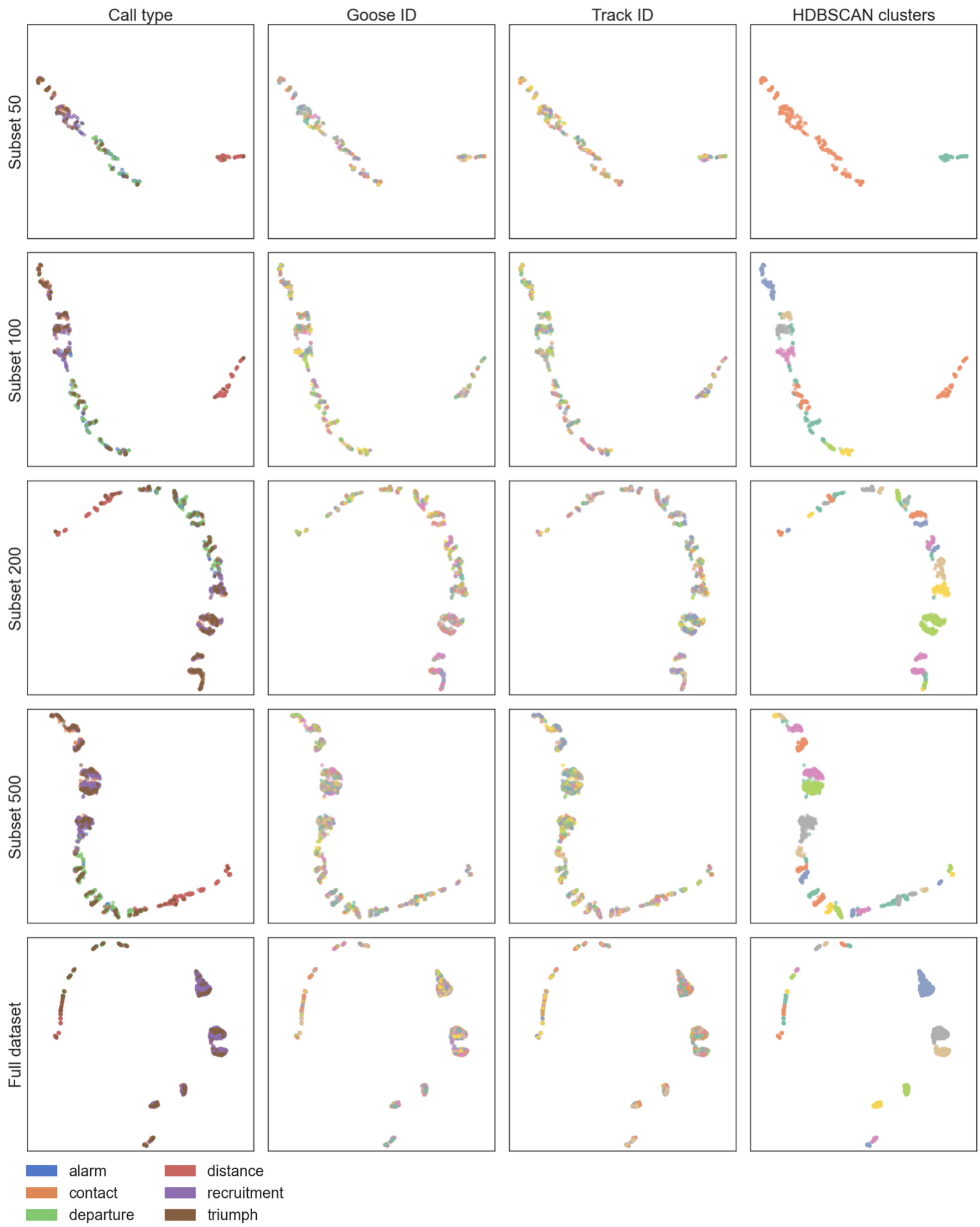

Figure S12 UMAP projections of LFCCs. Colours represent human-labelled call types (left column), Goose ID (second column), Track ID (third column), and clusters detected using HDBSCAN (right columns) with random colours for the different clusters. Subset sizes 100, 200, 500 and the full dataset generally led to more classes being predicted.

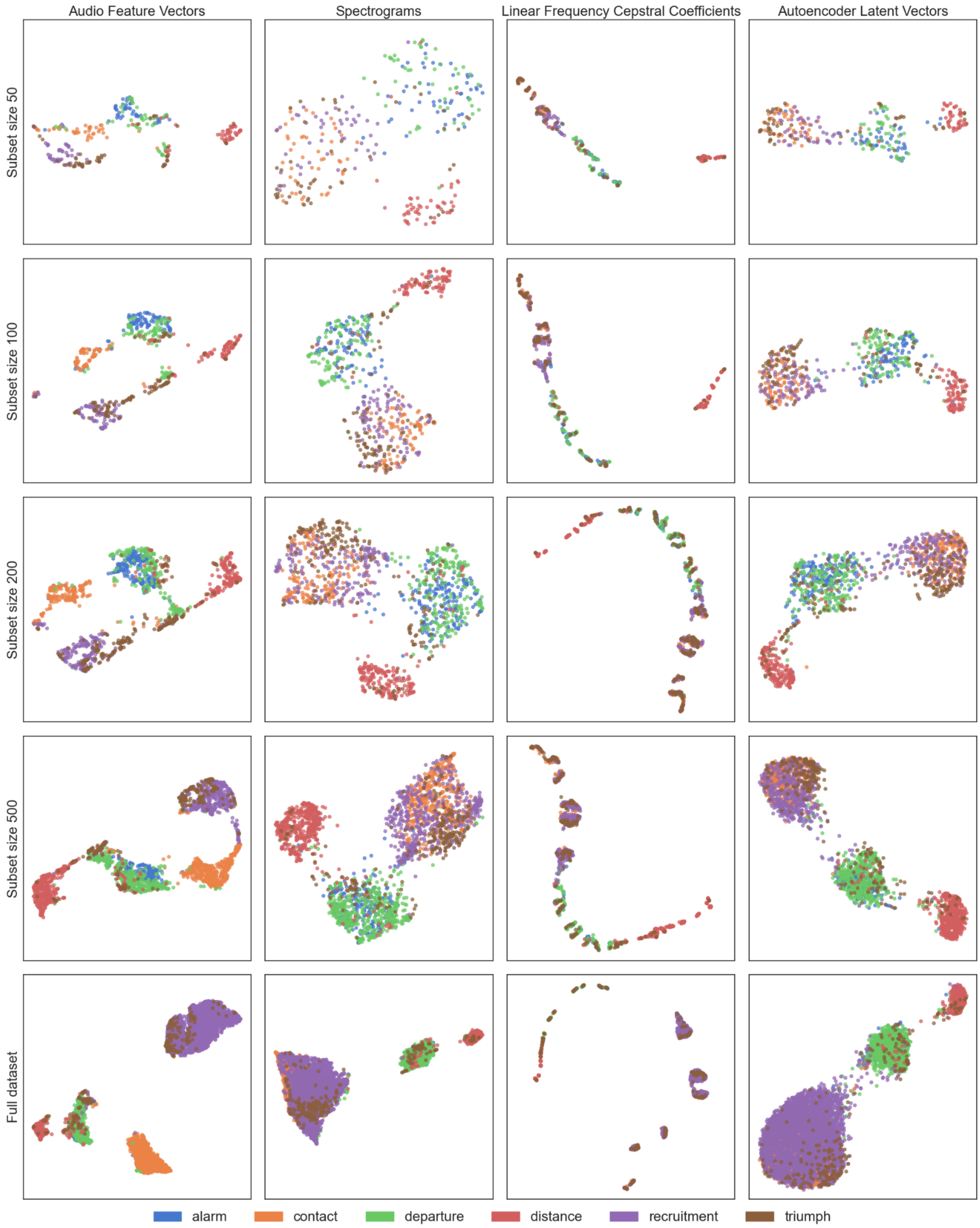

Figure S13 UMAP projections of audio feature vectors (left columns), spectrograms (second column), LFCCs (third columns) and autoencoder latent vectors (right columns) for different dataset sizes (rows). Colours represent human-labelled call types. Subset sizes 100, 200, 500 and the full dataset led to more classes being predicted for embeddings of LFCCs.

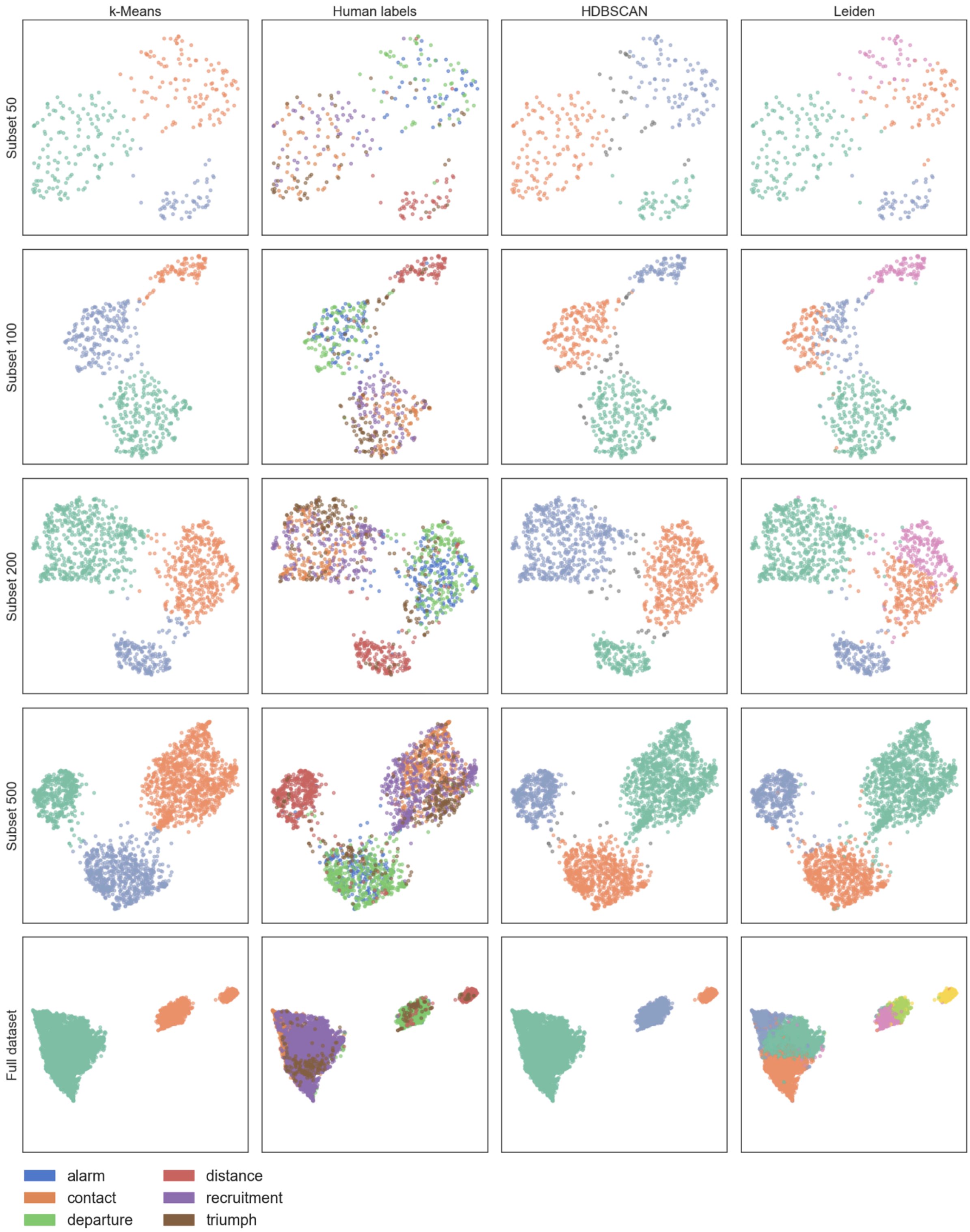

Figure S14 UMAP projections of spectrograms with different dataset sizes (rows). Examples of predicted classes in column one (kMeans), three (HDBSCAN) and four (Leiden). The second column shows the human labels.

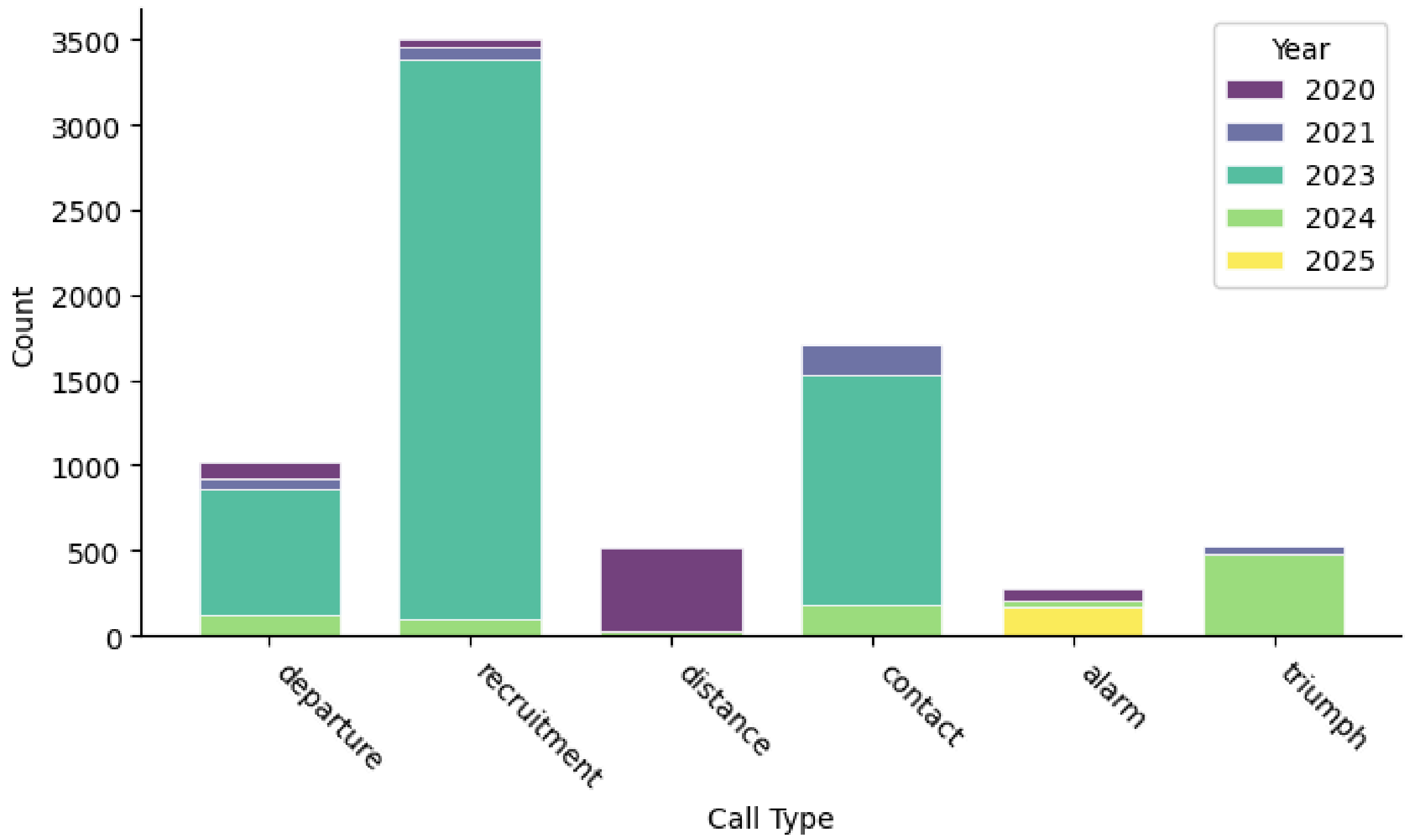

Figure S15 Number of collected call types per year.

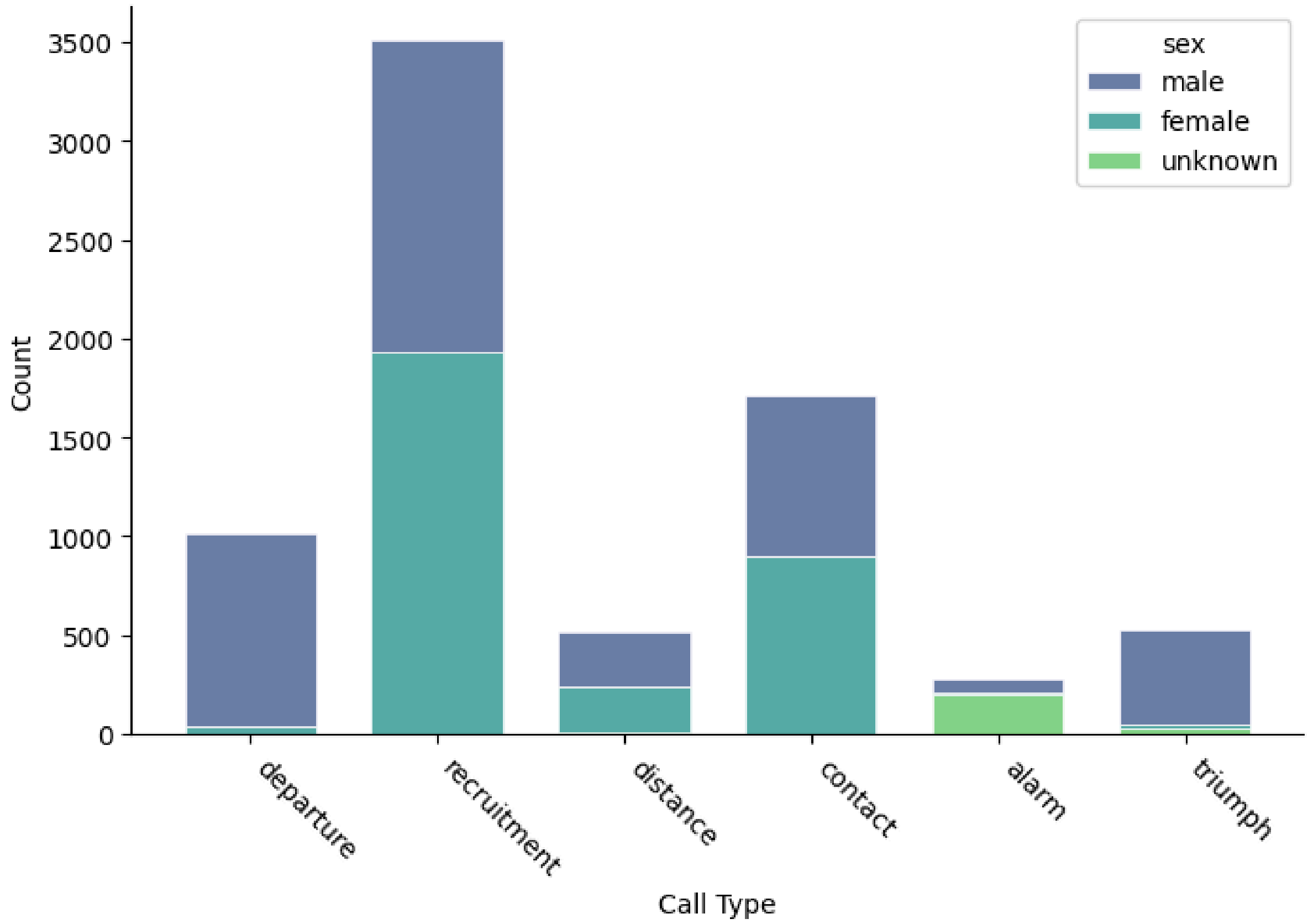

Figure S16 Distribution of sexes of the vocalizing individuals per call type category.

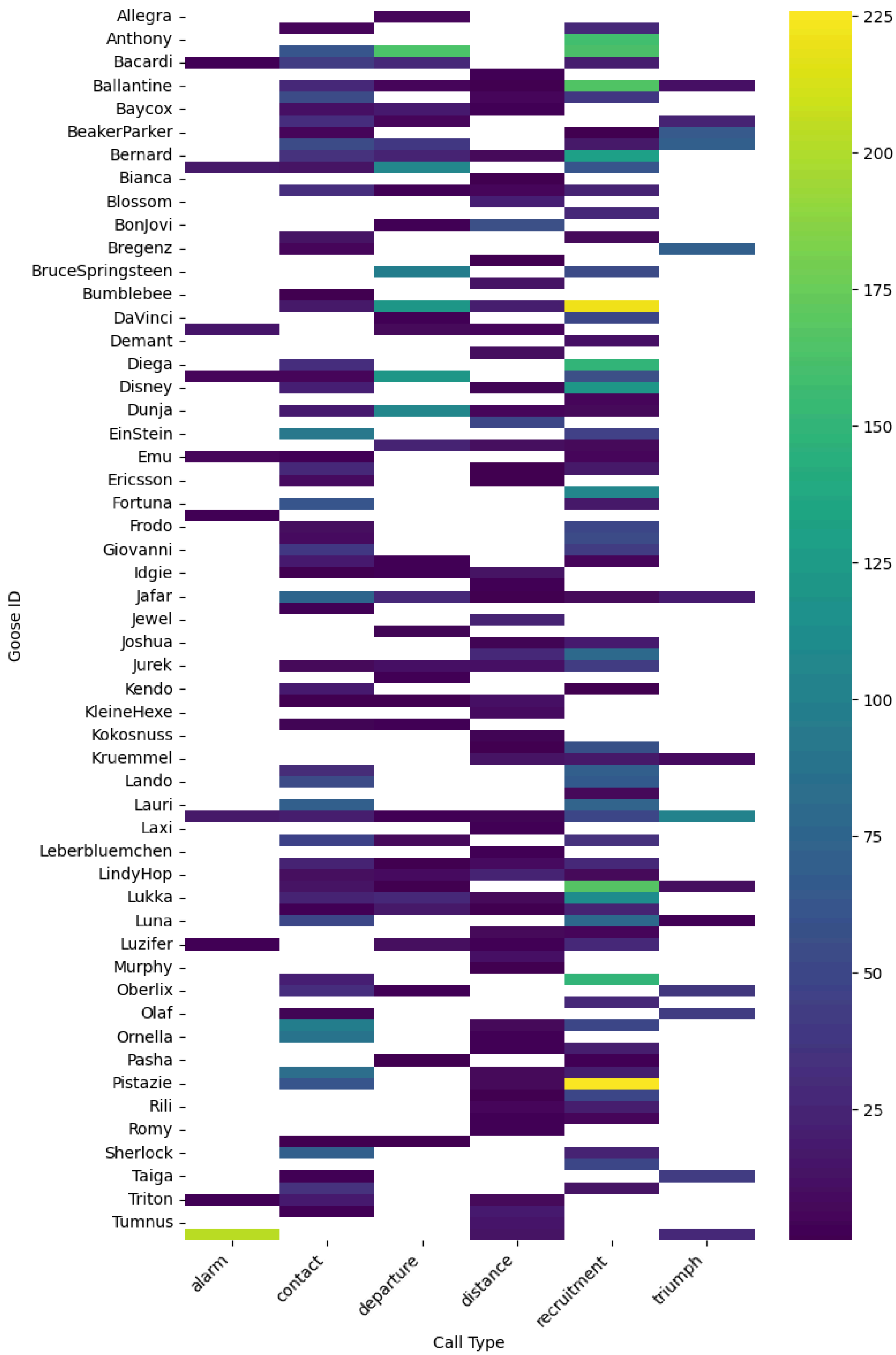

Figure S17 Data collected per goose. Columns represent call types while colors represent the number of calls collected per goose and category. Only every second label is displayed for the rows in the figure.

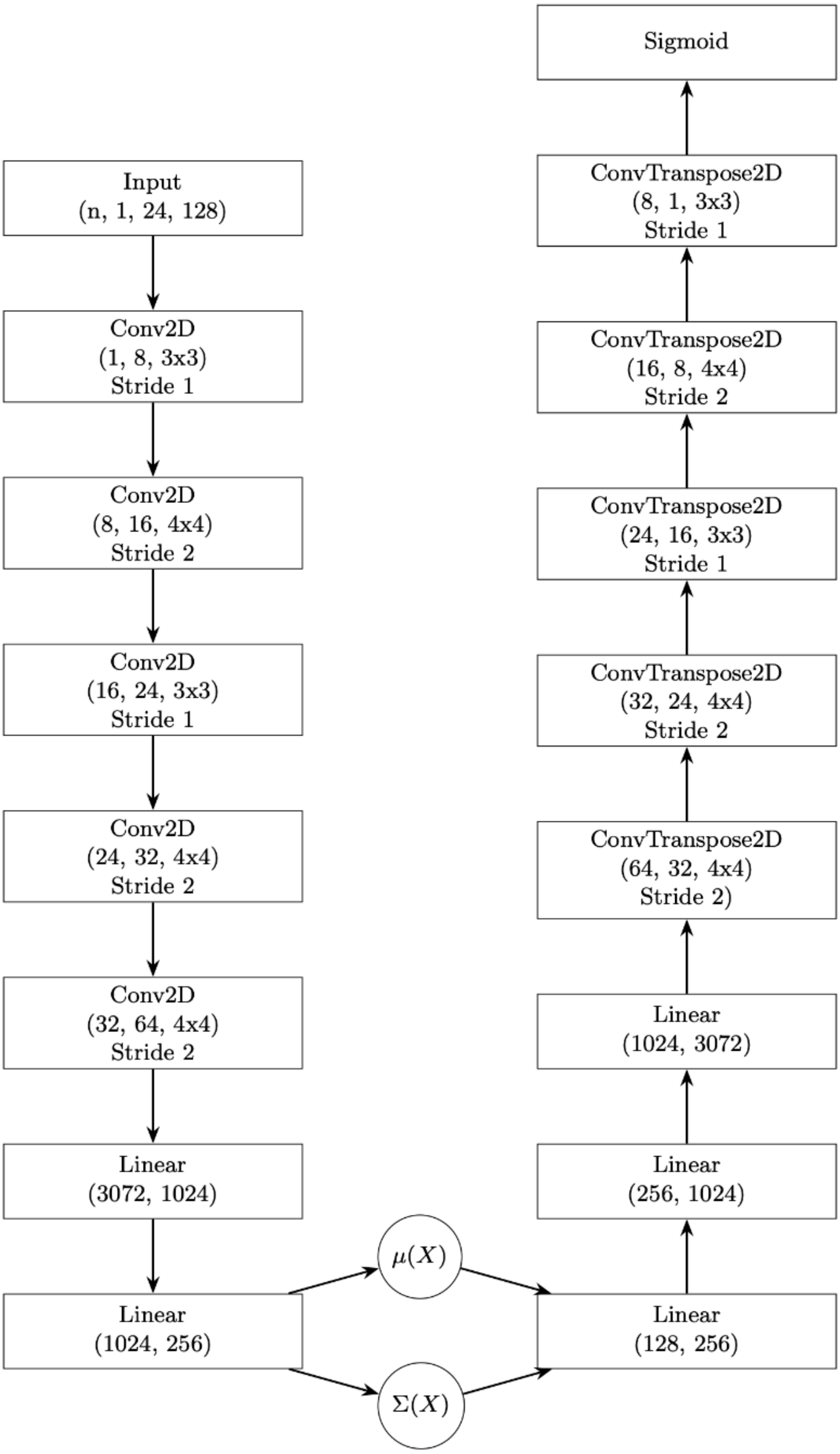

Figure S18 Layer design of the VAE with the encoder on the left and the decoder on the right. Every convolutional layer was followed by a batch normalisation layer. LeakyRELU was used as the activation function for all layer connections. The latent vector has a length of 128.
